# Three-Dimensional Electrochemical Sensors for Food Safety Applications

**DOI:** 10.3390/bios13050529

**Published:** 2023-05-08

**Authors:** Chi Zhang, Qingteng Lai, Wei Chen, Yanke Zhang, Long Mo, Zhengchun Liu

**Affiliations:** 1 Hunan Key Laboratory of Super Microstructure and Ultrafast Process, School of Physics and Electronics, Central South University, Changsha 410083, China; zc15022642253@163.com (C.Z.); laiqingteng100@163.com (Q.L.); ykzhang@csu.edu.cn (Y.Z.); 2 Department of Clinical Laboratory, Xiangya Hospital of Central South University, Changsha 410008, China; chenweiwei@csu.edu.cn; 3 Department of Cardiology, Xiangya Hospital of Central South University, Changsha 410008, China; diagnostics@126.com

**Keywords:** three-dimensional electrochemical sensors, 3D nanomaterial, food safety, components detection, synthesis and processing methods, detection of pathogens

## Abstract

Considering the increasing concern for food safety, electrochemical methods for detecting specific ingredients in the food are currently the most efficient method due to their low cost, fast response signal, high sensitivity, and ease of use. The detection efficiency of electrochemical sensors is determined by the electrode materials’ electrochemical characteristics. Among them, three-dimensional (3D) electrodes have unique advantages in electronic transfer, adsorption capacity and exposure of active sites for energy storage, novel materials, and electrochemical sensing. Therefore, this review begins by outlining the benefits and drawbacks of 3D electrodes compared to other materials before going into more detail about how 3D materials are synthesized. Next, different types of 3D electrodes are outlined together with common modification techniques for enhancing electrochemical performance. After this, a demonstration of 3D electrochemical sensors for food safety applications, such as detecting components, additives, emerging pollutants, and bacteria in food, was given. Finally, improvement measures and development directions of electrodes with 3D electrochemical sensors are discussed. We think that this review will help with the creation of new 3D electrodes and offer fresh perspectives on how to achieve extremely sensitive electrochemical detection in the area of food safety.

## 1. Introduction 

As society develops and people’s living standards rise, more and more food types are consumed as a daily diet [1,2]. In the meantime, foodborne infections are becoming a more significant threat to public health because of improper food handling and storage [3]. Therefore, it is crucial for practical reasons that pathogenic elements in food are quickly and sensitively detected, as well as hazardous compounds [4]. A variety of methods have been applied in the field of food detection, such as high-performance liquid chromatography (HPLC) [5], spectroscopy [6], spectrophotometry [7], capillary electrophoresis [8], etc. And the traditional methods for detecting foodborne pathogens are viable cell counting [9], plate separation [10], enzyme-linked immunosorbent assay (ELISA) [11], etc. However, these technologies have been limited in their applicability due to adverse elements, including expensive detection costs, sluggish response times, convoluted pre-processing and operation processes and poor detection accuracy [12].

Furthermore, especially in real samples, the detection results are often unsatisfactory under the influence of multiple similar interferents. Thus, a huge demand exists for detecting platforms with high sensitivity, high repeatability, and interference immunity. Electrochemical sensors are the most desirable detecting instruments because of their dependability, low detection limits, the outstanding response signal, and cheap assembly cost [13,14]. Furthermore, electrochemical sensors can detect and analyze the composition and content of substances to be measured by recording the changing electrical signal generated by the redox reactions between the electrode materials and the target materials. Therefore, electrochemical sensors can be easily utilized in food safety, medical testing, and water quality monitoring by simply synthesizing electrode materials that can undergo specific reactions with target materials [15,16].

The outcomes of electrochemical sensor detection are primarily represented by current, potential, and resistance changes depending on the various electrochemical signals [17,18]. And because electrochemical sensing is the most popular method of monitoring electric currents, this paper concentrates on it. For current indications, voltammetry and amperometry are the most common detection methods. Voltammetry comprises many forms, such as cyclic voltammetry (CV), differential pulse voltammetry (DPV), linear scanning voltammetry (LSV), etc. [19]. Amperometry differs from voltammetry in that it does not use a scanning potential during the detection process; instead, the change in current and time is recorded. Based on this, three-electrode electrochemical sensors can achieve detection not only in an electrolytic cell but also in real-time by creating tiny flexible electrodes or microfluidic devices [20]. So, constructing portable food detection equipment based on electrochemical sensing is promising.

With their distinctive physicochemical and surface charge characteristics, nanomaterials have recently made significant advancements in engineering and manufacturing [21,22]. In particular, nanomaterial-based electrochemical sensors are widely used to detect food safety [23]. Furthermore, compared to macroscopic materials, the larger surface area of nanostructured electrodes led to more significant electrocatalytic activity and detection response, achieving lower detection limits and faster response times [24]. Among them, three-dimensional (3D) nanomaterials outperform other materials like 0, 1 and 2-dimensional in adsorption capacity, mass transfer capacity, and site exposure capacity [25]. Therefore, 3D electrodes constructed from 3D nanomaterials are more likely to produce redox reactions with the measured ingredients. In addition, 3D electrodes can combine the advantages of one- and two-dimensional structures and even demonstrate electrochemical performance superior to their components [26,27].

At present, electrochemical sensors assembled by 3D electrodes are mainly divided into two categories, one is to build 3D arrays on the electrode surface, and the other is to modify the electrodes by using 3D structured nanomaterials [28,29,30,31]. The most common types of 3D nanomaterials are carbon materials and their derivatives, metal-organic frameworks (MOFs), metal oxides/hydroxides, alloys, and various composite materials [32,33]. However, 3D materials have several problems restricting their use in electrochemical sensing. The biggest concern is the electrode’s poor overall conductivity and propensity to aggregate when each structural unit is independent. Consequently, by means of metal particle doping, surface modification, and hole-making treatment, the electrical conductivity is considerably increased while keeping the original structure. Moreover, including 1D carbon nanotubes (CNTs), Ag nanowires, etc., can provide the same result [34]. Therefore, appropriate regulation of 3D electrode materials can improve electrochemical sensor detection accuracy, increase electron mobility, and fully exploit the benefits of 3D electrodes.

This article details the production, functionalization, and application of 3D electrodes in food safety electrochemical detection. 3D electrodes are based on carbon materials, MOFs, metal-oxides, alloys, and composites. The role of 3D structured electrodes-based electrochemical sensors in detecting ingredients, additives, heavy metal ions, emerging pollutants and bacteria is also explicitly shown. Lastly, the potential use of 3D electrochemical sensors for food safety applications is discussed. We think this review will broaden the reader’s ideas for guaranteeing food safety and help them understand 3D materials and electrochemical sensors built from 3D structured electrodes.

## 2. Preparation of Three-Dimensional Electrode

**Figure 1:**
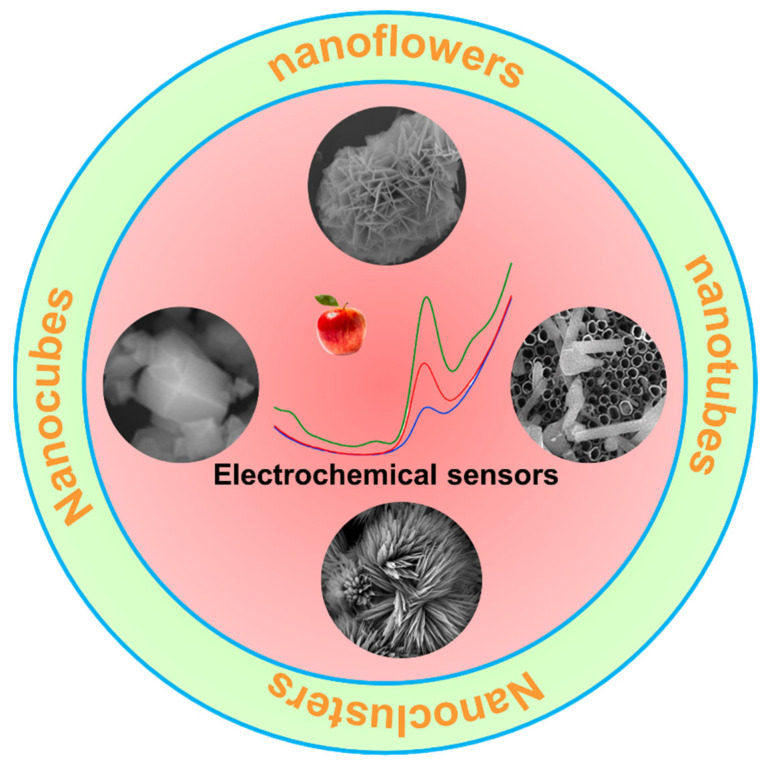
Several common 3D nanostructures prepared by hydrothermal method for electrochemical sensing (The inset shows the DPV response for different concentrations of diphenylamine at apple samples).

3D electrodes are usually made up of nanomaterials with 3D structures. In addition to physically combining 1D and 2D materials to obtain a mixed 3D structure, reasonable chemical synthesis methods are also important ways to get 3D nanomaterials with high electrochemical performance. Among them, the most representative technology is the hydrothermal (solvothermal) method [35,36]. This straightforward and effective method is frequently used to produce different 3D materials for electrochemical sensor applications because of the high purity of the synthesized material. Different reactions that can occur during hydrothermal processes include redox reactions, precipitation, decomposition, crystallization, etc. and regain the value of some undesirable reaction processes [37,38]. In particular, products obtained at high temperatures and pressure usually have a higher degree of crystallinity in crystallization reactions and therefore be applied directly after centrifugation, washing, and drying [39]. In addition, changing the reaction parameters such as the reaction temperature, the capacity of the reactor, and the number of reactants added can adjust the morphology, porosity, and size of the products, as well as the growth and loading can be accomplished with the assistance of surfactants or templates [40]. As shown in Figure 1, typical morphologies are synthesized by the hydrothermal method.

The electrodeposition technique also enables the synthesis and controlled preparation of 3D materials according to the growth pattern of crystals [41]. Electrodeposition, instead of hydrothermal techniques, help directly load active chemicals on conductive materials by electro-oxidation and electro-reduction reactions to strengthen their connection [42]. Compared to the electrolyte solution’s PH, temperature and ion concentration, the current density is the most critical factor controlling the morphology in the electrodeposition process. High current densities reduce ion diffusion leading to divergent growth of the material, while low current densities are more helpful in the nucleation of the material [43]. Consequently, the shape of the material can be accurately controlled by modifying the current magnitude of the external circuit. The short time usually required for electrodeposition is also a significant advantage. In some special cases, especially for 3D arrays of electrodes used in food detection, electrodeposition is the only viable option for preparing 3D arrays and uniformly decorated 3D arrays of nanosheets (NSs) or nanoparticles (NPs).

Chemical vapor deposition (CVD), as a bottom-up synthesis method, uses precursors that are heated to high temperatures and then driven by a gas stream to react with the active substrate [44,45]. Especially for synthesizing porous carbon materials with desirable mechanical properties and electrical conductivity, CVD technology can create electrodes with unique properties of three-dimensional structure [46,47,48]. For instance, 3D graphene produced by CVD offers high crystallinity and controlled layer thickness. On the other hand, 3D graphene with a curved shape also gives more exposed surfaces due to the weak coupling between the layers [49]. Likewise, the CNT obtained during the CVD reaction will be longer thanks to the water vapor flow cleaning [50]. Unfortunately, the CVD process often occurs at high temperatures and with catalysts. The way to regulate the material shape is to alter the temperature, pressure, and gas flow rate, which is a costly and dangerous process [51,52,53]. In addition, it is easy to produce toxic gas, which is a safety hazard, so CVD reaction is only selected under certain circumstances.

Template-assisted synthesis of 3D materials is a promising strategy widely used in electrochemical sensing in recent years [54]. In comparison to electrode materials, the creation and control of templates can frequently be accomplished in a single straightforward chemical step. Then, based on the limit and supporting properties of the template, the homogenous electrode materials can be produced without poor test result reproducibility due to the material’s varied sizes. Several templates are available, including soft, hard, and self-sacrificing templates used in assembly and synthesis [55]. These templates can restrict subsequent modification and loading to a specific region immediately surrounding them. Notably, as the template participates in the reaction, more ion diffusion channels are generated. The porosity and specific surface area considerably increase, all favorable to improve the material’s catalytic activity [56]. Typical template materials include nickel foam, zeolites, polypropylene microspheres, metal-organic frameworks, 2D MXene, and porous carbon [57,58,59]. Furthermore, depending on the properties and reaction principles of different templates, core-shell, spherical, rod and tube materials can be obtained by combining the above-mentioned three synthesis methods [60,61].

Another popular technique for preparing 3D materials is the sol-gel method, which is particularly useful for assembling multilayer films and 3D networks [62]. The fundamental idea is to uniformly disperse monomers, complexes or precursors through hydrolysis reactions and then fabricate 3D network nanoparticles or 3D materials through coupling and self-assembly processes [63,64]. Finally, the 3D solid materials with well-defined structures are obtained by subsequent treatment, such as drying and calcination. The purpose of the heat treatment is to eliminate any remaining solvents from the gelation process and to increase the strength of the bonds that have been formed. The following are the key benefits of the sol-gel method: (1) The reaction can be carried out under controlled circumstances at low-temperature thanks to the sol-gel process’s low synthesis temperature; (2) In particular for biosensing, the produced materials or nanocomplexes have higher purity and good affinity, and the favorable rheological features make them simple to load and adsorb enzymes; (3) By altering external factors during synthesis, such as temperature and pH, the shape and size of the final product can be accurately controlled [65,66]. Typical examples are that 3D flower-like neodymium molybdate and 3D bienzyme/Au NPs/multi-walled carbon networks were synthesized by sol-gel method and demonstrated satisfactory results in electrochemical detection of nitrofurantoin and acetylcholine [67,68]. It should be emphasized that sol-gel reactions typically take a while, and the final heat treatment step could damage the product’s structure or activity.

3D nanomaterials are also prepared using other synthesis methods, such as water bath heating, ultrasonic shaking, microwave-assisted preparation, etc. To select the most appropriate synthesis method, we must consider the material’s morphology, size, required quantity, and detection requirements. Besides preparing 3D nanomaterials, giving 3D structures or arrays to substrates is also an innovative idea for preparing 3D structured electrodes, such as using femtosecond laser [69], localized electrochemical deposition, and other emerging technologies that can build 3D electrodes for food safety detection as well.

## 3. Different Kinds of Three-Dimensional Materials and Three-Dimensional Structured Electrodes

**Figure 2:**
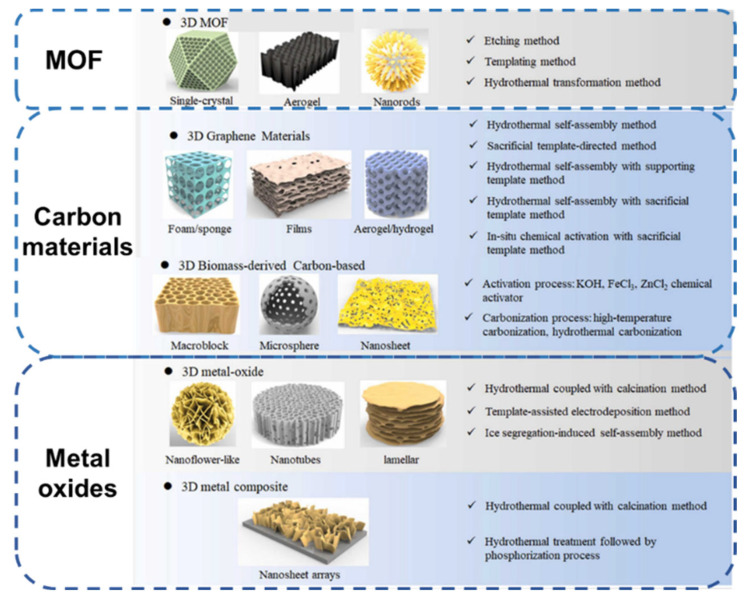
Basic composition and preparation method of 3D materials. Reprinted from [27] with permission from Elsevier.

3D electrode materials with high-dimensional structures have larger specific surface areas and more active reaction sites than 0, 1, and 2-dimensional materials. Moreover, the electrode’s 3D structure maximizes space utilization, minimizes “dead surfaces,” and guarantees enough porous channels in the laminate structure. Therefore, electrochemical sensors based on 3D structured electrodes have garnered much attention due to their great sensitivity and outstanding selectivity. Typical 3D materials comprise MOFs, carbon material and metal oxides, as shown in Figure 2.

### 3.1. Metal Oxides

**Figure 3:**
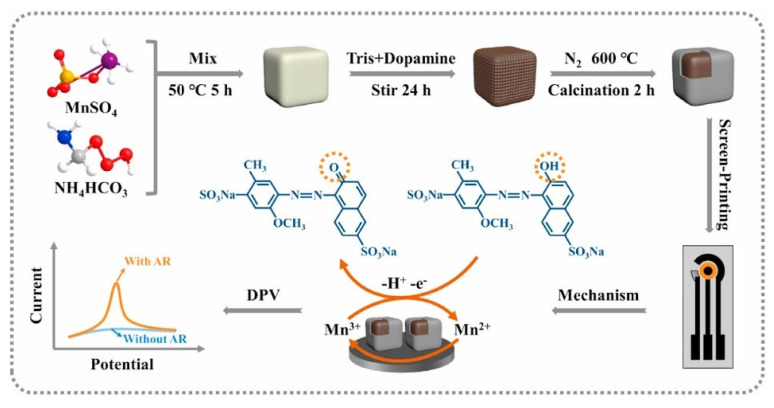
Schematic diagram of the synthesis of an electrochemical sensor based on the cubic structure of Mn_3_O_4_@C for the detection of AR. Reprinted from [77] with permission from Elsevier.

Structured and functionalized metal oxides have recently become the most promising materials for electrochemical sensing platforms. Due to their superior chemical properties, including excellent electrical and molecular properties, special surface charge properties, and large specific surface area, 3D-structured metal oxides offer substantial advantages in electrochemical sensing [70,71]. For example, compared to polymeric materials, metal oxides offer higher electrochemical stability and as opposed to carbon-based materials, not only have the same or greater energy storage capacities but can also display fast Faraday reactions between the electrode material and the molecule being detected within a defined potential window [71]. Generally, metal oxides used in electrochemical sensing applications should meet the following criteria: (1) metals have more than two oxidation states and can coexist with each other; (2) metal oxides can allow free ions to intercalate and departure from the lattice during redox reactions (such as interconversion of OH^−^ ↔ H_2_O). (3) The phase transition of the 3D structure is only reversible [72]. Among them, researchers have shown a lot of interest in the design of 3D morphology for metal oxides. In rational structures, there are more accessible interfaces that offer active sites for reactions and linkage sites obtained after modifications. And the functional groups and surface charges present at the interface are determined by the nature of the metal oxide. Currently, oxides of various metals such as Zn, Co, Fe, W, Ce, Sn and Ni have been synthesized to fabricate a variety of electrochemical sensors for the food safety field, including various pesticides, additives and ingredients that may be beneficial or harmful to humans depending on their morphology, structure, and size [73]. Common morphologies include nanoflowers, nanospheres, nanotubes, nanoclusters, and a variety of composite structures, and these 3D structures can enable sensors to achieve high sensitivity and wide linear ranges [74]. Figure 3 illustrates a highly stable Mn3O4@C based on a core-shell structure for electrochemical detection of Allura red (AR). However, oxides with nanoscale and porous structures are prone to side effects that cause a decrease in the transmission kinetics of the electrode due to an increase in grain boundaries. Meanwhile, many metal oxides have a wide band gap resulting in inadequate conductivity. Consequently, hybrid nanomaterials based on metal oxides were developed to address the shortcomings. For instance, combining 1D or 2D carbon materials can significantly increase the electrode’s conductivity, and electrodes having metal oxides and carbon materials can maximize each component’s benefits while improving detection abilities [75]. Another efficient method is the creation of multi-metal compounds. Compared to monometallic oxides, bimetallic oxides produce a synergistic effect with a considerably increased catalytic capacity [76]. Similar methods to improve the electrochemical properties of metal oxides include doping with precious metal particles, constructing heterojunctions and modulating the pore size structure. It is believable that metal oxides with 3D structures have excellent potential for electrochemical sensing uses.

### 3.2. MOFs

MOFs are 3D porous materials composed of inorganic agglomerates and organic binders, which can be strategically selected to improve detection performance [78,79]. Furthermore, MOFs are endowed with a homogeneous and uniform 3D structure, which allows them to have a tunable chemical function for a wide range of applications such as electrocatalysis, membrane separation, energy storage devices, nanocarriers and electrochemical sensing [80]. According to the available reports, The MOF synthesis process and the following modification procedure are the current research hot issues [81]. However, research on the synthetic mechanisms for the rapid and accurate synthesis of structurally intact and reasonable size MOFs for food safety analysis is still in its infancy [82,83]. Furthermore, the MOFs with the 3D structure are highly desirable in electrochemical sensing because their characteristics provide an enormous specific surface area and contribute more sites for aptamer attachment depending on their high porosity and abundant functional groups [84].

Due to unfavorable circumstances, such as an unstable MOF framework, uneven nucleation, and low conductivity, electrochemical sensors lose their sensitivity. Therefore, researchers are working to remedy this from the standpoint of the nucleation mechanism [85]. The coefficient of variation (COV) is an essential indicator of the homogeneity of MOF particle size. The satisfactory values of COV are less than 5%, but unfortunately, MOFs have only been gained with small amounts of canonical frameworks and frequently exhibit unfavorable huge size distributions (COV = 10–25%) [86]. Therefore, it is vital to lower the coefficient of variation in a suitable manner to maximize the performance of MOFs. The lamer model states that crystal growth and nucleation are asynchronous and that a rapid nucleation period is necessary to create homogenous [87]. To obtain homogeneous MOFs, for instance, ultrasound and microwave-based or traditional heating techniques are frequently employed in place of room temperature co-precipitation techniques. Haque et al. analyzed the nucleation process in the aforementioned three ways to demonstrate that MOFs produced by microwave heating and ultrasound are more homogeneous and that the mechanism of rapid nucleation has a beneficial impact on achieving uniform particle size [88].

Meanwhile, nanoreactor limitations with multiple solutions with various solubilities and coordinated modulation are both effective methods to achieve uniform nucleation. Typically, the *p*-perfluoro-ethylbenzoic acid addition to the reaction system, which regulates the nucleation rate, is the cause of the size homogeneity of MOF-5 discovered by Hermes et al. [89]. To break the Zn/Co-2-MIM bond to etch the outer surface with higher bond density, weak acids and metal-chelators are added, and on the other hand, the 2-MIM linkers are similarly protonated [90]. In parallel with this process and pH solution optimization, the shape of ZIF-MOF altered from a dodecahedron to a hollow cube. This demonstrates the positive impact on the uniformity of the final 3D MOF materials of rapid nucleation and higher reversible chemical manipulation. It should also be noted that the size of MOF particles for electrochemical sensing platforms is typically larger than 500 nm due to grafting, modification, or functionalization of the surface to increase conductivity [91].

Compared to smaller MOFs, choosing proper precursors and solvents, and increasing reaction concentration can aid in producing large-size MOF crystals [92]. At the physical level, selecting the right heating conditions or using microfluidics can assist in synthesising large-size 3D MOFs. The underlying strategy can be summarized as the growth rate and most nuclei react with each other by virtue of increasing the number of nucleation sites [93].

Additionally, because of their low conductivity, isolated 3D MOF materials only display their structural advantages in electrochemical sensing. The impedance to electron transport between the MOF blocks can be decreased using conductive materials to connect separated 3D nanoparticles, such as carbon materials, Ag nanowires, metal oxides, quantum dots and conductive polymers [94]. In 2017, Wang and co-workers synthesized a MOFs@PANI composite nanomaterial featuring Zr_6_O_32_ and 2-amino-terephthalate as a linker of 3D UiO-66-NH_2_ and tetrahedra and octahedra sharing a common triangular surface [95]. The electrochemical sensor manufactured using 3D MOF has a low detection limit of 0.3 μg L^−1^ and can consistently detect Cd^2+^ ions in the concentration range of 0.5–600 μg L^−1^. The synergy of the support structure supplied by 3D MOFs and the high conductivity brought by PANI gives the electrochemical sensor outstanding immunity and detection sensitivity. In this way, the sensitivity and selectivity of electrochemical sensors can be significantly improved by the large surface area and excellent conductivity of the structure. Utilizing metal NPs to modify the 3D MOF is another method of satisfying the demand for ultra-sensitive detection.

**Figure 4:**
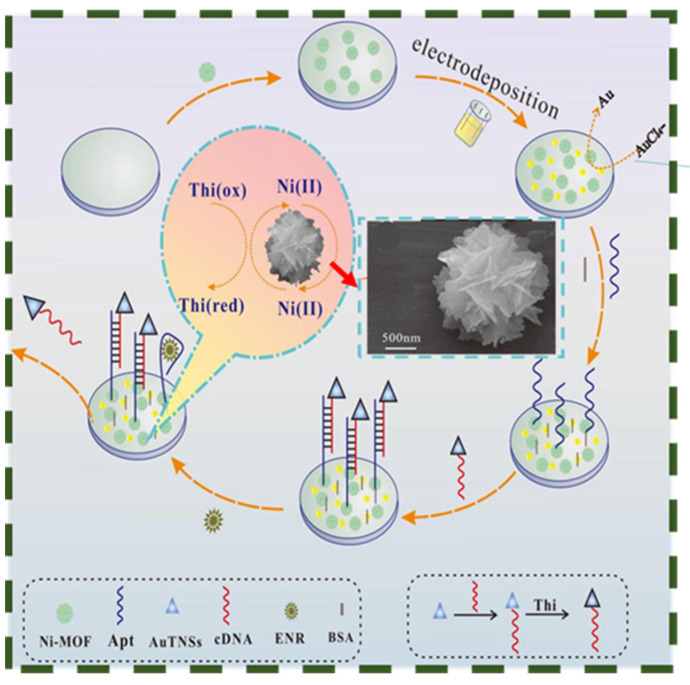
Schematic diagram of MOF-based aptamer electrochemical sensor for ENR detection (Insert shows the morphology and size of Ni-MOF). Reprinted from [96] with permission from Elsevier.

The lower overpotential obtained by adding noble metals facilitates electrocatalytic performance. Such as, Lv et al. had designed an electrochemical sensor based on Ni-MOF and Au NPs to detect enrofloxacin (ENR) [96]. As shown in Figure 4, modified electrodes had more aptamer anchoring sites, a bigger specific surface area, and faster transport rates. Comprehensive features included low detection limits, great repeatability and specificity because of distinctive structural properties and quick electron transfer. Due to their low cost and great efficiency, transition metals (Fe, Cu, Co et al.) are also employed to increase the conductivity of 3D MOF electrodes. To detect glutathione in complex environments, Xu et al. created a sensing platform that takes advantage of the modified Cu MOF with high conductivity resulting from the increasing number of redox sites by ferrocene creation [97]. Hybrid conductive silver paste, bimetallic co-modification, functionalized MOFs, and other similar techniques to boost the conductivity of 3D MOFs are all aimed at giving the electrode material a homogeneous 3D structure and a high electron transfer capability. Researchers have recently become interested in bimetallic MOFs containing two metal ions. Bimetallic MOFs exhibit a more stable 3D structure, greater efficiency and catalytic activity when compared to regular MOFs. So, utilizing MOF’s structural advantages to produce electrode materials with a large specific surface area and exceptional electronic properties is crucial when using them for electrochemical sensing [98].

### 3.3. Carbon Materials

To satisfy the need for simple sample preparation and efficient electrochemical sensing platforms, carbon materials have positioned themselves at the forefront of research due to their high electrical conductivity, ease of morphology tuning, and high mechanical strength [99,100,101]. Compared with single 0D carbon quantum dots (NPs with size less than 10 nm), 1D carbon nanotubes (diameter less than 10 nm, aspect ratio greater than 50), 2D graphene nanosheets and other carbon-based allotropes (lateral size greater than 100 nm but thickness of a few atomic layers), 3D carbon-based materials and carbon-based composites (stereoscopic structure on the micron scale) provide electrochemical sensors with a lower limit of detection and higher sensitive responses attributing to more active sites and larger specific surface area [102,103]. Therefore, significant efforts have been made to synthesize functionalized 3D carbon-based electrode materials. First, designing multi-walled carbon nanotubes to decorate the surface with functional groups such as carboxyl and hydroxyl can improve the catalyst’s dispersion [104]. For example, acidification can endow curved graphite flakes with a seamless hollow tubular 3D nanostructure. The chemical bond formed by sp2 and sp3 hybridization is the intramolecular s-bond with the partial characteristics of p orbital [105].

On the other hand, oxygen-containing functional group-grafted metal particles (such as Mn, Co, Ag.et) are also beneficial as sensing elements in electrochemical sensors [106]. Similarly, electrode materials were optimized by utilizing ionic liquids and chitosan-functionalized 1D carbon nanotubes [107,108]. Second, the integration of 1D carbon materials and 2D carbon materials into three-dimensional structures is a promising approach, and the resulting composites have larger specific surface areas, abundant active sites, and outstanding catalytic performance. And the unique structure also avoids the aggregation of nanosheets, which is conducive to the rapid movement of electrons. Third, the design and development of electrode materials are nanocomposites, including carbon nanotubes and 2d materials such as transition metal sulfides (MoS_2_), carbides (Ti_3_C_2_), graphene, etc. Taking full advantage of both materials and overcoming the aggregation of 2D materials, carbon nanotubes can help to establish stable 3-dimensional conductive layers, increasing mechanical stability and the connection of target molecules to the active materials [109,110].

**Figure 5:**
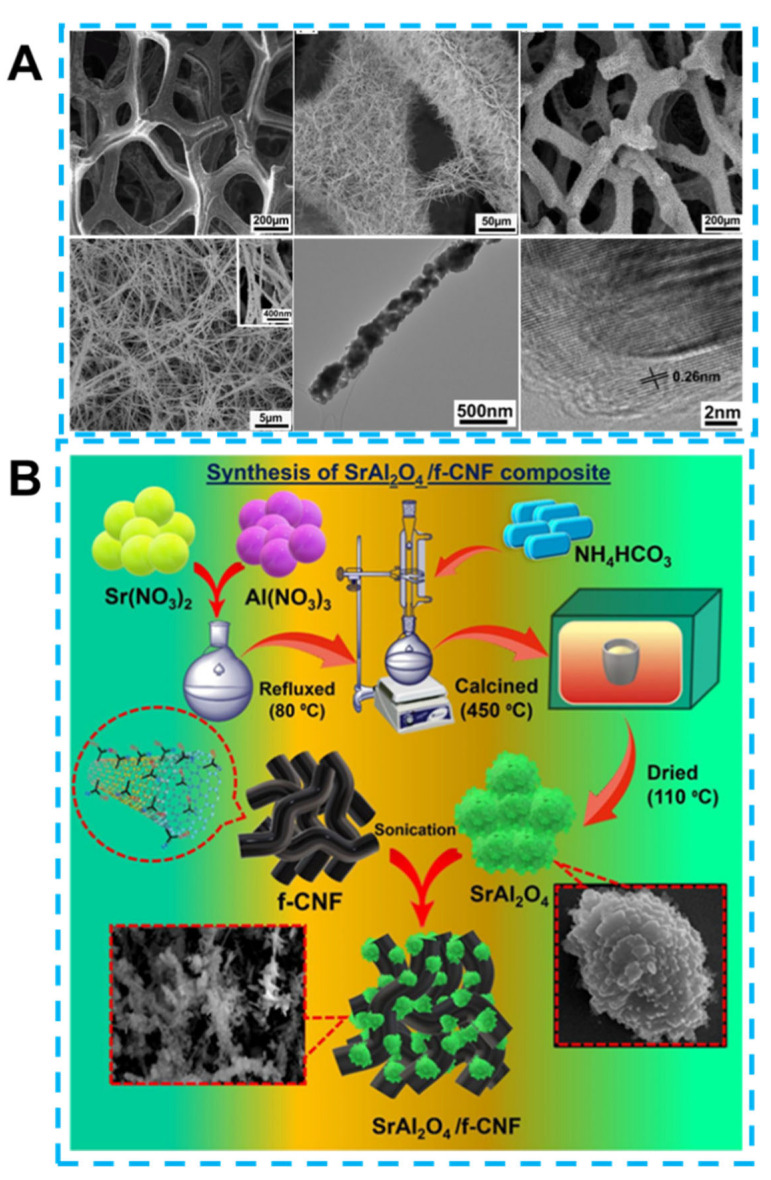
(**A**) Scanning electron microscopy images of 3D graphene and 3D graphene/Co_3_O_4_ composites at different magnifications and high-resolution transmission electron microscopy images. Reprinted from [103] with permission from the American Chemical Society. (**B**) Schematic diagram of synthetic SrAl_2_O_4_/f-CNF. Reprinted from [114] with permission from Elsevier.

Similarly, carbon nanotubes are crucial in connecting materials with 3D nanostructures. On the one hand, the synthesized nanocrystals were interconnected with a CNT-formed 3D network structure. On the other hand, Combining CNT embedded hollow nanocubes can also increase the contact area between the sensing material and the dielectric solution and promote electron transfer and electrocatalytic performance. The interconnected nanocubic morphology with a high surface area is the best choice for highly-sensitive electrochemical sensors considering uniformity and stability [111]. In addition, the synthesis of graphene-based 3-dimensional materials is a typical strategy. The three-dimensional structure determines the superiority of 3D graphene over 2D graphene due to the avoidance of aggregation and stacking of graphene sheets caused by strong π–π interactions. The synergy of this three-dimensional structure with the excellent inherent properties of graphene provides the continuous pathway for electron transport, increases the number of active sites, and protects the intrinsic high surface area and mechanical strength of graphene. These advantages are favored to improve the performance of electrochemical sensors [112]. As shown in Figure 5A, 3D graphene perfectly supports Co_3_O_4_ nanowires, and with the structural advantages, the composite materials enable precise enzyme-free detection of glucose. Carbon nanofibers are considered another perfect support material due to their remarkable aspect ratio (10–500 nm in diameter and Over 10 μm in length), favorable electron transfer kinetics. More notably, CNF with edge site defects is favorable for hyper heterojunction formation. Electrode consisting of petal-like material or layered double hydroxides (LDHs) material anchored to CNF is a 3D structure. Due to the synergistic effect of highly conductive CNF carriers and synthesized materials, as efficient electrocatalysts for sensing applications, high stability, the excellent performance of low detection limits, high sensitivity and selectivity are provided for the obtained sensors [113]. Figure 5B shows the construction of SrAl_2_O_4_/f-CNF-based Propyl gallate (PG) electrochemical sensors. Finally, electrochemical sensors based on 3-dimensional carbon materials such as carbon nanospheres and carbon nanocubes also exhibit high selectivity, low detection limits, and self-cleaning and recyclability advantages.

Compared to 2D carbon materials, 3D carbon matrix composites are used for electrochemical sensing due to their unique advantages. However, the negative impact of lattice defects and electronic properties on other materials should also be considered. In particular, the proportion of carbon material and the electrochemical interaction after the introduction of functional groups profoundly affect the sensitivity of electrochemical sensors.

### 3.4. Alloy and Perovskite-Type Oxides

**Figure 6:**
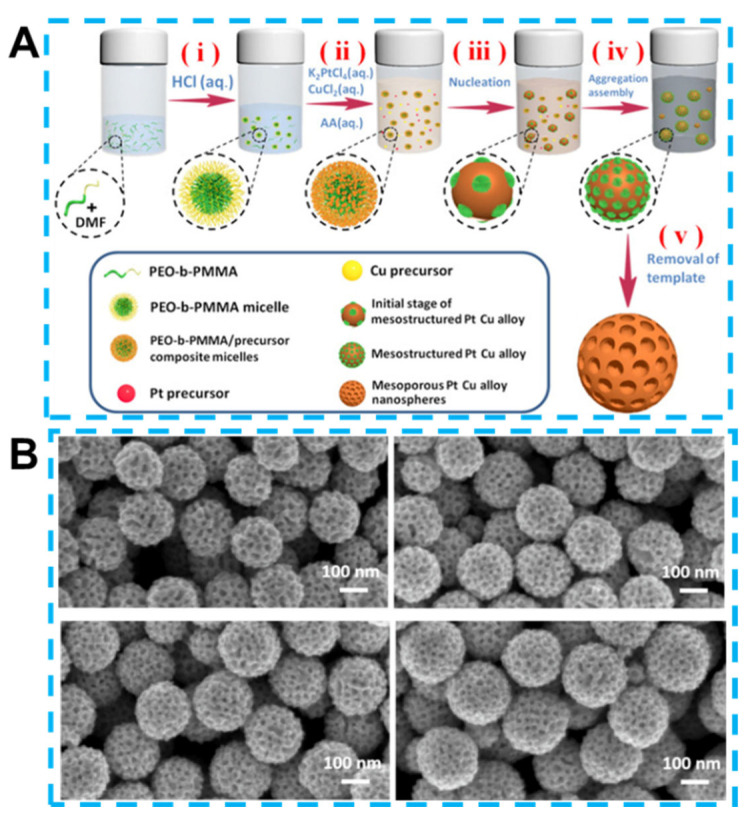
(**A**) Schematic diagram of the synthetic PtCu alloys. (**B**) Scanning electron micrographs of alloys with different ratios of Pt to Cu. Reprinted from [125] with permission from Wiley-VCH.

Alloys, a class of micro-structured materials, have recently been widely studied for their high catalytic efficiency and excellent electrical conductivity [115]. Compared to the metal nanoparticles frequently used in sensing systems, alloys with 3D structures have special advantages. For example, the self-supporting porous structure of the alloy reasonably avoids the aggregation of small metal particles while having a large specific surface area and many active sites [116]. Furthermore, the chemical reaction method used to create alloys typically does not need additional chemical assistance, such as surfactants, which guarantees the purity of the resulting alloy samples [117]. Chemical and electrochemical dealloying are two common synthetic techniques that work on the idea of etching the active component of the precursor under the right circumstances to produce a structured and uniform porous structure [118]. Chemical dealloying methods are relatively simple, while electrochemical methods make it easier to adjust the chemical composition. And due to alloy materials’ porous structure and durability above that of other nanomaterials, template-assisted synthesis is an excellent method [119]. Template-based synthesis of precursors allows for complete template removal in extreme media, and the resulting alloys will not be corroded by the medium and have all of the desired morphological and electrochemical characteristics. As illustrated in Figure 6A, the PtAu alloys prepared using soft templates have an intact shape and uniform pore distribution after thermal degradation. The change in particle size in Figure 6B is due to the different ratios of precursor Pt to Cu.

Similarly, electrochemical deposition and hydrothermal methods have synthesised alloys for electrochemical sensing applications [120,121]. However, it is essential to note that despite the potential of alloy materials for electrochemical sensing, particularly precious metals like Pd, Pt, and Au, developing more economical and cost-effective non-precious alloy materials is still necessary due to the limited cost of raw materials [122]. For example, Zhao et al. synthesized PtTi alloy and demonstrated that 3D nanoporous structures helped to achieve simultaneous ultra-sensitive detection of dopamine, ascorbic acid, and uric acid [123]. Moreover, Çelik et al. prepared a non-enzymatic hydrogen peroxide sensor based on 3D spherical TiNi alloys [124].

Furthermore, a specific class of perovskite-type oxides detect tiny molecules, including glucose, hydrogen peroxide, and others [126,127]. These materials have many oxygen vacancies and good electrical and catalytic properties. In addition, their optical and magnetic properties are more outstanding than those of other oxides. Based on this, Wang et al. and Govindasamy et al. designed 3D-structured perovskite-type nanomaterials for food safety detection, whose superoxo intermediates may cause distortions in the lattice in which they reside, thus showing irregular macroscopic cube construers leading to significantly enhanced electrocatalytic effects [128,129,130].

### 3.5. Three-Dimensional Structured Electrodes

While the synthesis of 3D materials described above is undoubtedly an excellent way to create 3D electrodes, due to the rapid development of processing conditions, the development of 3D arrays and stereoscopic planar electrodes are also novel approaches to increase the sensitivity of electrochemical sensors. Furthermore, femtosecond processing techniques, applied electric or magnetic field triggering techniques, 3D printing techniques, and photolithography have been widely used to process micro and nanostructures [131]. Therefore, the rational selection of 3D materials and the design of 3D electrodes will help enhance the detection capability of 3D electrochemical sensors.

## 4. Applications of 3D Sensors for Glucose Detection

**Figure 7:**
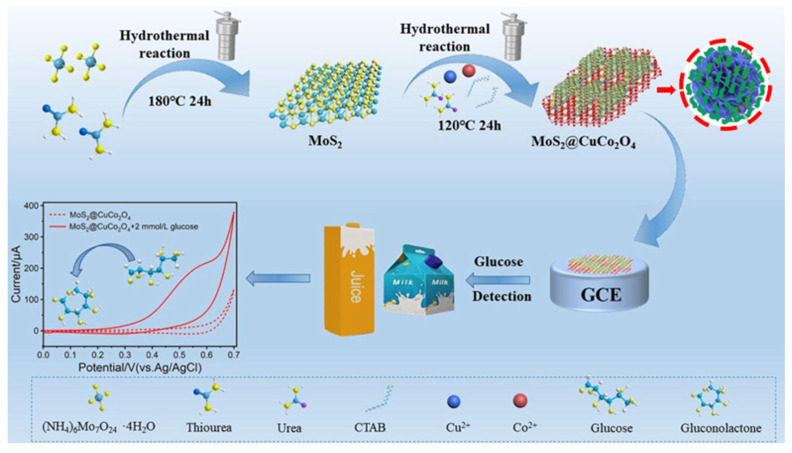
Schematic diagram of 3D MoS_2_@CuCo_2_O_4_-based glucose sensor preparation with MoS_2_@CuCo_2_O_4_ morphology concept illustrated in the inset and reprinted from [132] with permission from Elsevier.

The increasing incidence of diabetes has caused widespread concern among researchers year after year. The primary cause of this problem is overusing glucose in the diet. Electrochemical sensing techniques differ from colorimetric, optical approaches and SERC (surface-enhanced Raman scattering) because they are less expensive, more sensitive, and easier to build detection platforms. By using the catalytic oxidation of glucose, a class of non-enzymatic electrochemical sensors in electrochemical sensing avoid the loss of glucose oxidase activity brought on by the presence of oxygen in the environment, which would reduce detection sensitivity. Numerous 3D structured nanomaterials have been utilized as electrode materials for enzyme-free sensors because of their distinct formative benefits. Here, Wang et al. decorated two-dimensional MoS_2_ on CuCo_2_O_4_ using a hydrothermal technique to produce three-dimensional nanoflowers-like heterogeneous structures successfully employed to detect glucose in real-time [132]. The resulting MoS_2_@CuCo_2_O_4_ electrode materials exhibit superior sensing properties, mainly due to the 3D structure supported by MoS_2_ that prevents the stacking of nanosheets. As shown in Figure 7, the presence of more exposed active sites in this 3D-defined morphology relies on the large specific surface area of the material, which is also a crucial factor in determining glucose catalysis and detection. The sensors’ performance on this basis achieves an ultra-low detection limit of 0.5 μmol/L, sensitivity of 1303 μA mM^−1^ cm^−2^, an instantaneous response time of 2.1 s and a detection range of 0.5–393.0 μmol/L. Yan et al. prepared an N-doped carbon polyhedron with shell cores enriched with Co/CoO particles using the sacrificial template method [133]. This orthododecahedral shape with many meso- and macropores can speed up the electrochemical reaction and electron transport. The linear range of highly selective and sensitive enzyme-free assay for glucose was 0.01–16.65 mM, and the detection limit was 0.8 μM. In addition, MXene, a unique 2D material with high electrical conductivity and high hydrophilicity, is frequently employed in electrochemical research due to its surface-active functional groups, spatial support, and ability to act as a substrate material to support the nanosheet of layered double hydroxides (LDH) and metal oxides as a three-dimensional structure. A typical example is that Li and co-workers synthesized 3D MXene/NiCo-LDH as a catalytic material for electrochemical sensing using a simple hydrothermal method [134]. The 3D morphology of the composite and the synergistic effect of LDH and MXene are credited with the sensor’s low detection limit (0.53 μM), wide detection range (0.002 mM–4.096 mM), and quick response time (<3 s). The sensing platforms also have good selectivity and reproducibility, and they can consistently detect glucose in actual human blood. The rapid electron mobility in the 3D structure and the ease with which electrolytes diffuse in the three-dimensional structure determine the good detection capabilities.

## 5. Applications of 3D Sensors for Detections of Bioactive Food Components

The modern food industry must find green and healthy foods because the number of people who are currently unwell is rising owing to the irrational diet structures of contemporary people. The amount of beneficial ingredients in healthy food is an essential indicator of assessing food quality, so a safe and low-cost testing method is needed. Compared with other methods, electrochemical testing has the advantages of environmental protection, simple operation and high efficiency. For instance, Thenrajan et al. synthesized CoNiFe-ZIF microfibers via electrostatic spinning to detect green tea catechins (CTs) with antioxidant characteristics and increased physiological activity [135]. Compared to bare GCE, 3D porous microfibers loaded with NiCoFe-ZIF can be fully bound with CTs and have better sensing performance. The detection limits for the simultaneous detection of the three CT groups (epigallocatechin-3-gallate, epicatechin and epicatechingallate) corresponded to 45 ng, 8 ng and 4 ng, respectively. The linear detection range was 50 ng–1 mg. The antiviral and anti-inflammatory characteristics of luteolin (3′,4′,5,7-tetrahydroxyflavone), which are found in vegetables, have also caught the interest of experts. Tang et al. created an Ag-modified bimetallic CoNi-MOF at a temperature close to room temperature [136]. Ag-loaded flower-like microsphere structure can not only better promote the diffusion of electrolyte ions but also enhance the catalytic activity of luteolin. The Ag-CoNi-MOF-based sensors had a linear detection range of 0.002 to 1.0 μM and a detection limit of 0.4 nM.

## 6. Applications of 3D Sensors for Detections of Food Additives

**Figure 8:**
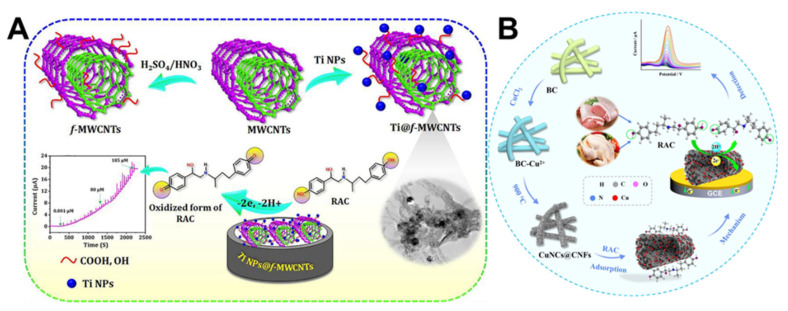
Schematic diagram of electrochemical sensing based on metal particles and carbon nanotubes at higher (**A**) and lower concentrations (**B**) of RAC. Reprinted from [143,147] with permission from Elsevier.

Smart use of food additives can increase consumer appetite but using them in excess is dangerous. As a result, Balram et al. synthesized a Ni-Co_3_O_4_ NPs/GO showing excellent response to the sulfonated azo dye Sunset Yellow (SY) in an electrochemical sensing platform [137]. Among them, the 3D structure of Co_3_O_4_ as ap-type semiconductor oxides with dual direct band gaps provides a clear and tunable structure. The addition of Ni nanoparticles to the Co_3_O_4_ lattice enhances conductivity. Finally, the hybridization with carbon-based materials significantly enhances the nanocomposite’s electrocatalytic abilities and electroactive surface area. Thanks to the synergistic effect, the linear range of the yellow sunset sensor was 0.125–108.5 μM, the detection limit was 0.9 nM, the sensitivity was 4.16 μA μM^−1^ cm^−2^, and the recovery rate was 96.16–102.56% in the actual detection of SY in various beverages and foods. In another research, Garkani Nejad et al. prepared MnO_2_ nanorods anchored graphene oxide (MnO_2_ NRs/GO) composites by hydrothermal method. As observed by FE-SEM, MnO_2_ NRs/GO displayed sheet-shaped and rod-like 3D mixed morphology [138]. The same structural benefits also allow composites to perform well in detecting SY. A detection limit of 0.008 μM was obtained, with a linear detection range of 0.01–115.0 μM. In addition, to Go, carbon materials with various morphologies and excellent electrical conductivity can be employed to detect SY sensitively. Using a one-step hydrothermal technique, Chen et al. synthesized hollow carbon spheres (HCS) and NiS composite-modified GCE for SY measurement [139]. The ability to transport electrons and detect SY is considerably enhanced by the synergistic action of NiS and HCS. The linear detection range of SY is 0.01–100 μM, and the detection limit is 0.003 μM. Joseph et al. combined WC with irregularly spherical FeMn-LDH to create composite materials with novel layered structures and successfully detected the antioxidant diphenylamine (DPA), which can protect food from spoilage during storage [140]. Due to the two materials’ deep hybridization and synergy, electrochemical sensors exhibited a wide linear range (0.01–183.34 μM) and a low detection limit (1.1 nM) that have been electrochemically confirmed. Ghalkhani et al. prepared a 3D hybrid structure of CoFe_2_O_4_@SiO_2_@HKUST-1 by self-assembly technique [141]. Under ideal circumstances, CoFe_2_O_4_@SiO_2_@HKUST-1 can significantly increase the oxidation of azaperone (AZN), frequently employed in animal husbandry as a muscle relaxant to improve meat quality. The electrochemical evaluation showed that AZN could be adsorbed and enriched on the surface of the CoFe_2_O_4_@SiO_2_@HKUST-1 modified electrode, and satisfactory detection results were obtained in the linear range of 0.05–10,000 nM with a detection limit of 0.01 nM. Since carbon material has good mechanical and conductivity capabilities, using 1D and 2D carbon materials in sensible designs is a smart way to increase the sensor’s performance. Xia et al. found that a molecularly imprinted proportional sensor constructed from a composite of carbon nanotubes, Cu_2_O NPs and Ti_3_C_2_T_x_ could achieve sensitive detection of the growth-promoting hormone diethylstilbestrol (DES) [142]. The accordion structure of Ti_3_C_2_T_x_ can adsorb large amounts of loaded Cu_2_O NPs by electrostatic force, while CNT can improve the sensor’s sensitivity. The electrochemical sensors can detect DES in a wide concentration range of 0.01 to 70 μM, with good linearity and a very low detection limit (6 nM). Similar work was the functionalization of multi-walled carbon nanotubes (MWCNTs) using metal particles by Keerthi et al. [143]. Strong interactions caused the spherically positively charged Ti particles to grow on tubular MWCNTs. This typical tubular shape was interconnected and exhibited remarkable electrocatalytic effects on the Ractopamine (RAC) animal feed additive, which was widely utilized in the livestock business. At slightly higher concentrations, the sensing system had a wide detection range (0.01–185 μM) with a detection limit of 3.8 nM. And at even lower concentrations, Lei et al. constructed copper and carbon composites with different morphologies than Xia et al. to detect RAC [144]. As illustrated in Figure 8, the Cu nanoclusters anchored on biogenic carbon constituted a 3D fiber network topology structure, so a more negative potential and higher activity were obtained. Therefore, this electrochemical sensors for RAC detection can perform ultra-sensitive detection at very low concentrations, with detection characteristics including a detection range of 2–850 nm, an ultralow trace detection limit of 0.05 nm and a sensitivity of 1641 μA μM^−1^ cm^−2^. Ma et al. prepared nanocarbon-supported NiFe_2_O_4_ by impregnation and carbonization techniques, and they used it to detect clenbuterol that had negative impacts on people. This neural network-like carbon structure-based electrochemical sensors have wide detection ranges of 10^−7^–10^−5^ M and 7 × 10^−11^–10^−7^ M, a low detection limit of 3.03 × 10^−12^ M and abilities to detect clenbuterol in real urine [145]. Vanillin (VN) is a typical flavor enhancer frequently added to meals and drinks. However, the addition of VN must be rigorously limited. To detect VN in food, Radhakrishnan et al. proposed an electrochemical method based on different morphologies of CuS [146]. By comparing the detection efficiency of different 3d structures synthesized by the solvothermal method, the CuS hexagonal-based sensors detected VN in the 0.1–46.5 µM with a detection limit of 53 nM. And the sensitivity was also excellent (0.85 µA/µM cm^−2^)

## 7. Applications of 3D Sensors for Detections of Food Preservatives

Another typical food additive is nitrite, and too much of it might result in tissue hypoxia. To precisely monitor nitrite in food, He et al. developed an Ag nanoparticle (Ag NPs)-graphite carbon electrochemical sensor platform using femtosecond laser technology [69]. Thanks to the laser pulse treatment to create a complete 3D flower-like micro-nano structure and mixed with Ag NPs, which increases the conductivity of the composite, this electrochemical sensing exhibited a wide detection range (1–4000 × 10^−6^ M) and a low detection limit (0.117 × 10^−6^ M). Additionally, the sensor allows for highly sensitive dopamine detection and has remarkable reproducibility. Using a more cost-effective hydrothermal technique, Kogularasu et al. designed g-C_3_N_4_/Bi_2_S_3_ composites to detect nitrite [147]. Depending on 3D sea urchin architecture, g-C_3_N_4_/Bi_2_S_3_ can serve as the active centre and enrich NO^2^ for a stronger electrochemical signal. Meanwhile, g-C_3_N_4_/Bi_2_S_3_ exhibited a larger peak current than bare GCE and single component modified GCE. As a result, this detection platform for nitrite achieved an extremely wide linear detection range of 0.001–385.4 μM and a detection limit of 0.4 nM. Another material that proved effective in detecting nitrite was metal oxide. As ZnO has a strong binding energy of 60 meV and a wide band gap of 3.37–3.44 eV, it is thought to be the most easily tunable and structurally well-defined material among metal oxides. Cheng et al. designed uniformly dispersed spherical ZnO nanomaterials, which can be more likely to adsorb negatively charged No^2−^ due to the zeta potential of +28.4 mV and RSD of 2.29% and utilized as electrochemical sensors for the detection and analysis of nitrite [148]. This 3D spherical ZnO-based sensing system was verified by chronoamperometry tests to have optimal linear detection ranges of 0.6 μM–0.22 mM and 0.46 mM–5.5 mM, a detection limit of 0.39 μM and a sensitivity of 0.785 μA μM^−1^ cm^−2^. Similarly, Somnet et al. obtained homogeneous spherical PdNPs@MIP using the molecularly imprinted technique to detect nitrosodiphenylamine, a derivative of nitrite [149]. PdNPs@MIP modified graphene electrodes for nitrosodiphenylamine detection with linear detection ranges of 0.01–0.1 μM (r^2^ = 0.996) and 0.1–100 μM (r^2^ = 0.992), the detection limit of 0.0013 μM. Direct growth of 3D arrays on the surface of GCE is also an idea to improve sensor performance. Lu et al. electrodeposited polymer polypyrrole (PPy) nanocones on the GCE surface and then loaded Co particles onto PPy [150]. Modified 3D electrodes demonstrate sensitive detection of nitrate encompassing a very wide linearity range of 2–3318 μM, the sensitivity of 2.60 μA μM^−1^ cm^−2^ accompanied with LOD of 0.35 μM. Han and co-workers reported a 3D flower-like MoS_2_ composite modified with silver nanoparticles to decorate glassy carbon electrodes to detect the food additive butylated hydroxyanisole (BHA) [151]. Morphological analysis revealed that the silver particles are uniformly covered in 3D flowers-like MoS_2_, resulting in more active reaction sites and a greater capacity for electron transport. The improved electrode offers a broad linear detection range (1 × 10^−9^–1 × 10^−4^ mol/L) for BHA under ideal conditions, with incredibly low detection limits (7.9 × 10^−9^ mol/L). Especially in actual food detection, the distinct MoS_2_ structure given this sensing platform remarkable selectivity and 103% recovery. Another widely used food preservation agent is tert-butylhydroquinone (THBQ), and long-term THBQ intake might result in various bodily discomforts. Therefore, to meet the monitoring of THBQ residues in the food industry, using MOF-based electrochemical sensors is an effective method. For instance, a strategy to synthesize porous nitrogen-doped TiO_2_-carbon composites using MOF was reported by Tang et al. [152]. Its huge specific surface area and porous structure considerably improve the catalytic efficacy of GCE modified with TiO_2_/NC for THBQ. THBQ was quantified by the octahedral TiO_2_/NC with a wide linear range of 0.05–100 μM and a detection limit of 4 nM. Luo et al. obtained transparent ZnO/ZnNi_2_O_4_@porous carbon with a polyhedral structure by pyrolysis of Ni-ZIF-8, a typical MOF material. Then the amine aldol condensation process produced ZnO/ZnNi_2_O_4_@porous carbon@COF_TM_ nanocomposite [153]. GCE modified with this nanomaterial made THBQ more susceptible to oxidation, so better detection results with a linear range of 47.85 nM–130 μM and a detection limit of 15.95 nM were attained. At the same time, the sensing platform can also detect paracetamol (PA) in the linear range of 48.5 nM–130 μM, with a detection limit of 12 nM. Balram et al. prepared the Co_3_O_4_ NRs/FCB (functionalized carbon black@Co_3_O_4_ nanorods) composite structure of spherical FCB uniformly spread over the rod-shaped spinel Co_3_O_4_ using the sonochemical method [154]. Screen printed carbon electrodes (SPCE) was modified with Co_3_O_4_ NRs/FCB composites for the ultra-sensitive detection of THBQ with a broad detection range (0.12–62.2 μM), an extremely low detection limit (1 nM) and an ultra-high sensitivity of 7.94 μA μM^−1^ cm^−2^. For a visual representation, the performance of electrochemical sensors based on 3D structured electrodes for detecting different food additives is summarized in Table 1.

## 8. Applications of 3D Sensors for Detections of Emerging Pollutants 

### 8.1. Detection of Heavy Metal Ions

Heavy metal ions persist in the natural environment for a long time, and their long-term accumulation and non-degradation can seriously endanger human health. Electrochemical detection has advantages over conventional approaches, including high sensitivity, low cost, and ease of use. And then a crucial aspect in determining the effectiveness of the sensing is the design and tuning of functionalized nanomaterials used as electrodes. For instance, Ru et al. demonstrated an octahedral GO/UiO-67@PtNPs nanocomposite employed to detect As (Ⅲ) in Chinese Herbal Medicines [155]. The uniform dispersion of Go/UiO-67 provided many sites for As (Ⅲ) immobilization while Pt is electrocatalytic to As (Ⅲ). Thus the nanocomposite exhibits excellent electrical performance. The electrochemical sensor based on this nanocomposite displayed outstanding properties for As (Ⅲ) detection concluding the detection limit (0.42 nM) and linear response range (2.7–40 nM). Liu et al. also synthesized Cu@carbon nanoneedles in the same sea urchin shape based on the conductivity of carbon materials [156]. This unique 3D structure can effectively immobilize a large amount of auxiliary DNA hybridized with Hg^+^ ions and achieve circular amplification of the signal with the assistance of exonuclease III. The electrochemical sensor fabricated by this strategy achieved satisfactory results in detecting Hg^+^ in terms of ultra-wide linear detection range (10 fM–10 μM) and low detection limits (7 × 10^−6^ ppm).

### 8.2. Detection of Pesticides

**Figure 9:**
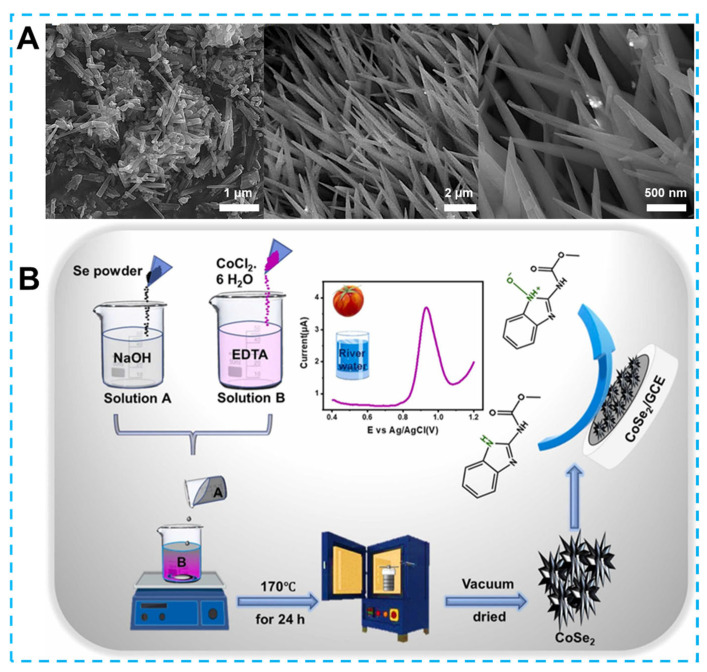
(**A**) Scanning electron micrograph of the typical 3D nanograss-like structures and (**B**) schematic diagram of 3D sea urchin-like o-CoSe_2_ synthesis. Reprinted from [159,160] with permission from Elsevier.

In the field of agriculture, the use of pesticides properly can increase agricultural yields. However, excessive pesticide residues can permanently harm people’s health and the ecosystem. Therefore, the creation of straightforward and affordable electrochemical sensors for detecting pesticide residue is urgently needed. First, Ganesan et al. developed an electrochemical sensor based on 3D neodymium molybdate since the toxicity of methyl parathion (MP, organophosphorus pesticides) can seriously interfere with the nerve center [157]. The Nd_2_Mo_3_O_9_ (NdM) with 3D flower-like structures constructed from 2D nanosheets possess large specific surface areas and hierarchical porous structures as determined by BET analysis. Thus, porous flower-like NdM nanosheets (pf-NdM NSs) as an electrode material provide additional active sites and interconnected permeable channels for rapid charge transport. The excellent catalytic activity of pf-NdM NSs leads to a low detection limit of 5.7 nM and a sensitivity of 1.88 µA µM^−1^ cm^−2^, allowing this electrochemical sensor to respond in the range of 0.5–300 μM. In particular, the absence of large fluctuations in peak current indicates that the electrochemical sensing platform allows consistent and reliable detection of MP at tens of times the concentration of interfering molecules such as uric acid, catechol, hydroquinone etc. For similar organophosphorus pesticides, ratiometric electrochemical immunosensors for the detection of phoxim were fabricated, as reported by Su et al. [158]. DNA tetrahedras modified by 3D Au NPs can be excellent carriers for phoxim monoclonal antibodies and improve target signals. Therefore, the detection range of this sensor was between 0.1 and 30 μg/L, and the detection limit was 0.003 μg/L. Mahmoudi-Moghaddam et al. prepared Pt-doped nickel cobaltite utilizing a one-step hydrothermal method [159]. Using this grass-like nanomaterial as an electrode material, an electrochemical sensor was constructed to detect carbendazim (CBZ, a fungicide widely used in agriculture). Under ideal circumstances, the sensor used to detect CBZ had a linear range of 0.03–140 μM and a low detection limit of 0.005 μM. As shown in Figure 9, further research led Tamilalagan and his associates to develop a three-dimensional urchin electrochemical sensor based on cobalt diselenide (o-CoSe_2_), improving CBZ detection performance [160]. The specific surface area and catalytic activity of the pure GCE electrode are enhanced by the three-dimensional structure of CeO_2_. In addition to a detection range of 0.01–382.43 μM and a lower detection limit of as low as 1.69 nM, the sensor can detect and maintain high accuracy, interference resistance and repeatability in actual water and tomato samples. Based on the same structural features, Akilarasan and co-workers reported the design and fabrication of similar sea urchin-like bimetallic nanocomposites strontium sulfide and bismuth sulfide (SrS/Bi_2_S_3_) [161]. Using shape-homogeneous and adjustable morphology SrS/Bi_2_S_3_ to detect the hazardous pesticide maleic hydrazide, the sensors display optimal detection range (0.01–104 µM and 104–814 µM) and detection limit (1.8 nM). In addition, because the sea urchin-like structure of the SrS/Bi_2_S_3_-modified electrode surface has more catalytic sites, the electrochemical platform demonstrated ideal recoveries and reproducibility in detecting potato and river water samples. Yamuna et al. used graphene to oxidize Ni_2_Te_3_O_8_ to obtain Ni_3_TeO_6_ [162]. The performance of Ni_3_TeO_6_ composites was investigated using electrochemical analysis to detect metribuzin (MTBZ) as another common herbicide. The experimental results revealed that the composite material composed of inhomogeneous spheres and large rods had high sensitivity (1.454 µA µM^−1^ cm^−2^), wide detection range (0.01–161.36 µM) and low detection limit (1.62 nM) for MTBZ detection.

### 8.3. Detection of Antibiotics and Drugs

Antibiotics accumulated in food are a further pollutant that threatens food safety. As reported by Manjula et al., hexagonal plates of NiO/ZnO were used for the rapid and accurate detection of chloramphenicol [163]. Due to the hexagonal plate-like morphology of the NiO/ZnO-modified electrode, which is more favorable for the adsorption of chloramphenicol, the peak current was substantially higher than that of the unmodified electrode. The detection limit is calculated to be 0.07 μM, and the detection range is 0.05–418.5 μM with good stability and immunity to interference. For the same detection of the antibiotic nitrofurantoin (NFT), Li et al. built a detection platform with Co_2_Mo_3_O_8_/MoS_2_ as the electrode mediator [164]. Co_2_Mo_3_O_8_/MoS_2_@CC comprised Co-MOF, MoS_2_ nanoflowers and carbon cloth. Its 3D hybrid structure was packed with more unsaturated sites provided by MoS_2_ and benefited from the synergistic interaction of the three, which increases structural stability and electrocatalytic performance. So, the electrochemical sensor for NFT detection can respond in the range of 100–700 μM because of the heterostructure of Co_2_Mo_3_O_8_/MoS_2_@CC, which had a low detection limit 11.9 nM and a sensitivity of 27.6 μA μM^−1^ cm^−2^. Drugs pose a serious threat to society, so it’s crucial to swiftly and precisely identify their drug residues in discharges. Zou et al. presented Fe_3_O_4_@MIPs based on molecularly imprinted polymers synthesized using microwave-assisted [165]. Ketamine, as the main component of K powder, was well detected by electrochemical sensors made of homogenous Fe_3_O_4_@MIPs spheres throughout a large linear range of 1.0 *×* 10^−12^–4.0 × 10^−4^ mol L^−1^ with a limit of detection as low as 8.0 × 10^−13^ mol L^−1^. Also, using highly selective MIPs, Roushani et al. created a GCE comprised of MIPs, aptamers, and Cu_2_O cubes. The well-defined angular cubic structure gives the modified GCE additional sites for aptamer loading, leading to outstanding results in detecting aflatoxin [166]. The linear detection range and detection limits were calculated and defined as 50.0 pg L^−1^–3.5 ng L^−1^ and 3.5–40.0 ng L^−1,^ and 12.0 pg L^−1^.

## 9. Applications of 3D Sensors for Detections of Foodborne Bacteria

**Figure 10:**
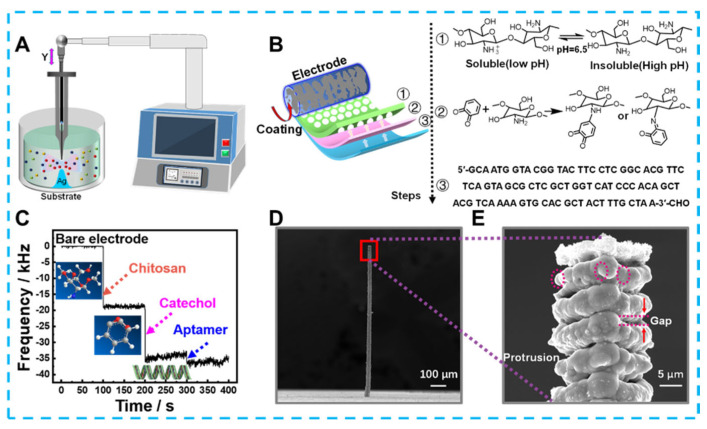
(**A**) The schematic diagram for preparing Ag micro−nano−pillar electrodes, (**B**) process of modifying electrodes, (**C**) Mass change of electrodes after each modification step recorded by microbalance, (**D**) scanning electron microscope image of 3D Ag microelectrode and (**E**) local morphology.

Developing emerging technologies for the electrochemical detection of bacteria could significantly reduce foodborne illnesses caused by bacterial contaminants. For example, Feng et al. prepared an electrochemical immunosensor based on Fe_3_O_4_@graphene composites to detect *Salmonella* in milk [167]. The 3D complex could immobilize monoclonal antibodies by loading a large amount of Au-NH bonds due to the growth of almost spherical Fe_3_O_4_ on a 2D graphene substrate, and Au NPs that also provided Au-NH could amplify an electrical signal. Electrochemical detection of *Salmonella* by these 3D composites was performed within the range of 2.4 × 10^2^ and 2.4 × 10^7^ cfu/mL with the detection limit of 2.4 × 10^2^ cfu/mL. In addition, composite 3d materials containing graphene have shown high sensitivity in electrochemical sensing without immunoreactivity. For example, Fatema et al. focused on designing materials to prepare and apply Cu-Sn codoped BaTiO_3_-G-SiO_2_ (CuSnBTGS) to detect *Rhizoctonia stolonifer* [168]. CuSnBTGS had many mesopores and an ideal surface area, which N_2_ adsorption—desorption isotherms had confirmed was due to its mixed cubic sphere and lamellae structure. The rapid migration of electrons on the CuSnBTGS surface also resulted in a linear relationship between the sensing *Rhizoctonia stolonifer* concentration in the range of 50–100 μL, and the detection limit was 0.50 μL. Along with synthesizing 3D structured nanomaterials, designing 3-dimensional microstructured electrodes can also optimize the detection effect. As an example of a typical optimized planar electrode shown in Figure 10, Lai et al. created Ag micro-nano-pillars that resembled multilayer cakes using local electrochemical deposition [169]. The uneven surface of these nano-pillars formed a localized electric solid field that caused Cl^−^ ions within the bacteria to flow out. And the Cl^−^ ions were used to label bacteria in the pseudo-capacitive electrochemical sensor constructed using Ag micro-nano-pillars. Compared to flat structures and smooth Ag columns, Ag micro-nano-pillars with rough surfaces was more capable of inducing Cl^−^ ions, as confirmed by COMSOL simulations. Therefore, the sensor achieves ultra-sensitive detection for *Staphylococcus aureus* in the linear range of 1–10^5^ CFU mL^−1^ with a detection limit as low as1 CFU mL^−1^ and maintains specificity in the presence of nine other species of bacteria. In addition to the traditional colony count, detecting biomarkers is an essential indicator of bacterial viability and damage. Zheng et al. used hydrangea-shaped MoS_2_ modified by AuPd nanoparticles to design electrochemical aptamer sensors [170]. Meanwhile, porous Co-MOF and MCA 3D hybrid materials offer a clearly defined structure and reliable electrochemical characteristics. In particular, the structural advantage of a large specific surface area provides many sites for methylene blue (MB) loading. More active surfaces also offer the potential to penetrate many nucleotide chains driven by π–π stacking interaction and electrostatic adsorption interaction. Based on the above advantages, electrochemical aptasensors were developed to detect adenosine triphosphate (ATP) biomarkers. With reasonable use of the 3D structure of the active material and the signal label, not only is signal amplification achieved, but the detection range of 10 pM–100 μM and detection limits as low as 7.37 × 10^−10^ μM are the ideal embodiment of performance.

## 10. Conclusions and Future Outlooks

This review summarises the application of three-dimensional (3D) electrochemical sensors in food safety. Firstly, many common synthetic techniques for producing 3D nanomaterials are presented, with each technique’s benefits and drawbacks. Then the effects of different synthesis methods on the morphology, size, electrical conductivity, and specific surface area of the prepared materials under different conditions are discussed with the aim of providing reasonable ideas in the design, preparation and modification of 3D nanomaterials. Secondly, different 3D materials are rationally classified, which helps to select the most suitable materials for evaluating the electrochemical detection of different types of substances. Additionally noted are the distinct benefits of 3D structured electrodes produced by micro- and nano-processing technologies for 3D electrochemical sensing. Thirdly, exemplary works illustrate that 3D electrochemical sensors have a great deal of potential for applications in the detection of components, additives, emerging pollutants, and bacteria in food, thanks to 3D structures with unique shapes and a significant number of exposed active surfaces. Finally, the ability of the carefully tuned 3D electrochemical sensors to detect real samples was also underlined, which is of greater relevance to food safety and human health.

Despite the valuable research results that have been achieved, there are still some limitations and challenges in building 3D structured electrodes for electrochemical sensings, such as: (1) A part of the nanomaterial preparation process is complex, the cost of raw materials is high, and the repeatability is poor, which severely reduces the range and frequency of sensor use; (2) The binding principle between the obtained material and the target substance is not clear, resulting in poor selectivity of the sensor and poor targeting of the synthesized material; (3) The construction of sensing platforms is often based on laboratory environments, and portability and real-time detection capabilities need to be improved. For the problems mentioned above, future solutions and ideas can be summarized as follows: first, an effective solution is to synthesize more promising materials or to modify existing ones. For example, the conductivity of the obtained material can be increased by adding conductive materials or synthesis costs are reduced by the rational use of non-precious metal raw materials. Another idea is redesigning the electrode using the available machining tools to optimize the planar electrode. Secondly, theoretical calculations are used to determine the materials’ electron distribution and binding energy. Finally, the obtained material is finely characterized by X-ray absorption near edge structure (XANES) and spherical aberration electron microscopy. Based on the characterization results, the synthesis conditions are reasonably optimized. At last, according to the existing research base and the needs of society, combining the sensing system with portable devices like ARM to convert electrical signals to digital signals and finally realizing real-time detection is the focus and direction of future research. With continuous efforts, we have reasons to believe that electrochemical sensors based on 3D electrodes will make more progress in the field of food safety in the future.

## Figures and Tables

**Table 1:** Performance of electrochemical sensors for food additives based on different 3D electrodes.

Sensor Materials	Structure or Morphology	Analyte	Range of Detection	Detection Limit	Refs.
CoFe_2_O_4_@SiO_2/_HKUST-1	approximate octahedron	AZN	0.05–10,000 nM	0.01 nM	[141]
MIP/CNT/Cu_2_O NPs/Ti_3_C_2_Tx	3D composite structures	DES	0.01–70 μM	6 nM	[142]
WC@FeMn-LDH	spheres	DPA	0.01–183.34 μM	1.1 nM	[140]
Ti@f-MWCNTs	3D composite structures	RAC	0.01–185 μM	0.0038 µM	[143]
CuNCs@CNFs	3D network structures	RAC	0.002–0.025 μM	0.05 nM	[144]
Co_3_O_4_ NRs/FCB	nanorods	TBHQ	0.12–62.2 μM	1 nM	[154]
TiO_2_/NC	octahedrons	TBHQ	0.05–100 μM	4 nM	[152]
ZnO/ZnNi_2_O_4_ @porous carbon@COF_TM_	polyhedrons	TBHQ	47.85 nM–130 μM	15.95 nM	[153]
MoS_2_/Ag NPs-CS	3D-flowers	BHA	1 × 10−9^−1^ × 10^−4^	7.9 nM	[151]
Ni-Co_3_O4 NPs/GO	3D composite structures	SY	0.125–108.5 μM	0.9 nM	[137]
MnO_2_ NRs/GO	nanorods	SY	0.01–115.0 μM	0.008 μM	[138]
NiS@HCS	hollow spheres	SY	0.01–100 μM	0.003 μM	[139]
NiFe_2_O_4_–NPCs	3D network structures	CLB	10^−7^–10^−5^ M and 7 × 10^−11^–10^−7^ M	3.03 × 10^−12^ M	[145]
CuS	hexagons	VN	0.1–46.5 μM	53 nM	[146]
Co/PPy	nanocones	nitrite	2–3318 µM	0.35 µM	[150]
g-C_3_N_4_/Bi_2_S_3_	3D sea-urchins	nitrite	0.001–385.4 μM	0.4 nM	[147]
ZnO	spheres	nitrite	1.9–800 μM and 1080–5900 μM	0.89 μM	[148]
LaAlO_3_@GO	3D composite structures	nitrite	0.01–1540.5 µM	0.0041 µM	[128]
PdNPs@MIP	microspheres	nitrite	0.01–0.1 μM and 0.1–100 μM	0.0013 μM	[149]
Ag NPs-Graphite Carbon	3D micro/nano morphologies by femtosecond Laser	nitrite	1–4000 × 10^−6^ M	0.117 × 10^−6^ M	[69]

## Data Availability

Not applicable.

## References

[1] Wang Z., Liao F., Guo T., Yang S., Zeng C. (2012). Synthesis of crystalline silver nanoplates and their application for detection of nitrite in foods. J. Electroanal. Chem..

[2] Rassaei L., Marken F., Sillanpää M., Amiri M., Cirtiu C.M., Sillanpää M. (2011). Nanoparticles in electrochemical sensors for environmental monitoring. TrAC Trends Anal. Chem..

[3] Kaur H., Siwal S.S., Chauhan G., Saini A.K., Kumari A., Thakur V.K. (2022). Recent advances in electrochemical-based sensors amplified with carbon-based nanomaterials (CNMs) for sensing pharmaceutical and food pollutants. Chemosphere.

[4] Curulli A. (2022). Recent Advances in Electrochemical Sensing Strategies for Food Allergen Detection. Biosensors.

[5] Moudgil P., Bedi J.S., Aulakh R.S., Gill J.P.S., Kumar A. (2019). Validation of HPLC Multi-residue Method for Determination of Fluoroquinolones, Tetracycline, Sulphonamides and Chloramphenicol Residues in Bovine Milk. Food Anal. Methods.

[6] Llamas N.E., Garrido M., Nezio M.S.D., Band B.S.F. (2009). Second order advantage in the determination of amaranth, sunset yellow FCF and tartrazine by UV–vis and multivariate curve resolution-alternating least squares. Anal. Chim. Acta.

[7] Pourreza N., Fat’hi M.R., Hatami A. (2012). Indirect cloud point extraction and spectrophotometric determination of nitrite in water and meat products. Microchem. J..

[8] Ryvolová M., Táborský P., Vrábel P., Krásenský P., Preisler J. (2007). Sensitive determination of erythrosine and other red food colorants using capillary electrophoresis with laser-induced fluorescence detection. J. Chromatogr. A.

[9] Rajapaksha P., Elbourne A., Gangadoo S., Brown R., Cozzolino D., Chapman J. (2019). A review of methods for the detection of pathogenic microorganisms. Analyst.

[10] Liu S., Zhao K.X., Huang M.Y., Zeng M.M., Deng Y., Li S., Chen H., Li W., Chen Z. (2022). Research progress on detection techniques for point-of-care testing of foodborne pathogens. Front. Bioeng. Biotechnol..

[11] Chae W., Kim P., Hwang B.J., Seong B.L. (2019). Universal monoclonal antibody-based influenza hemagglutinin quantitative enzyme-linked immunosorbent assay. Vaccine.

[12] Karimi-Maleh H., Arotiba O.A. (2020). Simultaneous determination of cholesterol, ascorbic acid and uric acid as three essential biological compounds at a carbon paste electrode modified with copper oxide decorated reduced graphene oxide nanocomposite and ionic liquid. J. Colloid Interface Sci..

[13] Fu J., An X., Yao Y., Guo Y., Sun X. (2019). Electrochemical aptasensor based on one step co-electrodeposition of aptamer and GO-CuNPs nanocomposite for organophosphorus pesticide detection. Sens. Actuators B Chem..

[14] Choudhary M., Siwal S., Nandi D., Mallick K. (2016). Single step synthesis of gold–amino acid composite, with the evidence of the catalytic hydrogen atom transfer (HAT) reaction, for the electrochemical recognition of Serotonin. Phys. E Low-Dimens. Syst. Nanostruct..

[15] Zhou Y.L., Yin H.S., Li J., Li B.C., Li X., Ai S.Y., Zhang X.S. (2016). Electrochemical biosensor for microRNA detection based on poly (U) polymerase mediated isothermal signal amplification. Biosens. Bioelectron..

[16] Xu J., Liu Y., Huang K.-J., Hou Y.-Y., Sun X., Li J. (2022). Real-Time Biosensor Platform Based on Novel Sandwich Graphdiyne for Ultrasensitive Detection of Tumor Marker. Anal. Chem..

[17] Nikolaus N., Strehlitz B. (2008). Amperometric lactate biosensors and their application in (sports) medicine, for life quality and wellbeing. Microchim. Acta.

[18] Li T., Shang D., Gao S., Wang B., Kong H., Yang G., Shu W., Xu P., Wei G. (2022). Two-Dimensional Material-Based Electrochemical Sensors/Biosensors for Food Safety and Biomolecular Detection. Biosensors.

[19] Ronkainen N.J., Halsall H.B., Heineman W.R. (2010). Electrochemical biosensors. Chem. Soc. Rev..

[20] Heinze J. (1993). Ultramicroelectrodes in Electrochemistry. Angew. Chem. Int. Ed. Engl..

[21] Maduraiveeran G., Sasidharan M., Ganesan V. (2018). Electrochemical sensor and biosensor platforms based on advanced nanomaterials for biological and biomedical applications. Biosens. Bioelectron..

[22] Liu J., Li T., Zhang H., Zhao W., Qu L., Chen S., Wu S. (2022). Electrospun strong, bioactive, and bioabsorbable silk fibroin/poly (L-lactic-acid) nanoyarns for constructing advanced nanotextile tissue scaffolds. Mater. Today Bio.

[23] Valiev R. (2002). Nanomaterial advantage. Nature.

[24] Piscitelli A., Pennacchio A., Longobardi S., Velotta R., Giardina P. (2017). Vmh2 hydrophobin as a tool for the development of “self-immobilizing” enzymes for biosensing. Biotechnol. Bioeng..

[25] Qiu B., Xing M., Zhang J. (2018). Recent advances in three-dimensional graphene based materials for catalysis applications. Chem. Soc. Rev..

[26] Zou C.E., Yang B., Bin D., Wang J., Li S., Yang P., Wang C., Shiraishi Y., Du Y. (2017). Electrochemical synthesis of gold nanoparticles decorated flower-like graphene for high sensitivity detection of nitrite. J. Colloid Interface Sci..

[27] Li X., Lu S., Zhang G. (2023). Three-dimensional structured electrode for electrocatalytic organic wastewater purification: Design, mechanism and role. J. Hazard. Mater..

[28] Zhu D., Zhen Q., Xin J., Ma H., Tan L., Pang H., Wang X. (2020). A free-standing and flexible phosphorus/nitrogen dual-doped three-dimensional reticular porous carbon frameworks encapsulated cobalt phosphide with superior performance for nitrite detection in drinking water and sausage samples. Sens. Actuators B Chem..

[29] Park S.-W., Yun E.-T., Shin H.J., Kim W., Lee J., Kim D.-W. (2021). Three-dimensional construction of electrode materials using TiC nanoarray substrates for highly efficient electrogeneration of sulfate radicals and molecular hydrogen in a single electrolysis cell. J. Mater. Chem. A.

[30] Bai H., Shi G. (2007). Gas Sensors Based on Conducting Polymers. Sensors.

[31] Kokulnathan T., Sakthi Priya T., Wang T.-J. (2019). Surface Engineering Three-Dimensional Flowerlike Cerium Vanadate Nanostructures Used as Electrocatalysts: Real Time Monitoring of Clioquinol in Biological Samples. ACS Sustain. Chem. Eng..

[32] Kokulnathan T., Wang T.-J. (2019). Synthesis and characterization of 3D flower-like nickel oxide entrapped on boron doped carbon nitride nanocomposite: An efficient catalyst for the electrochemical detection of nitrofurantoin. Compos. Part B Eng..

[33] Wei Z., Zhu W., Li Y., Ma Y., Wang J., Hu N., Suo Y., Wang J. (2018). Conductive Leaflike Cobalt Metal-Organic Framework Nanoarray on Carbon Cloth as a Flexible and Versatile Anode toward Both Electrocatalytic Glucose and Water Oxidation. Inorg. Chem..

[34] Zhang L., Tao H., Ji C., Wu Q., Wang X., Wu Y. (2022). Sensitive and direct electrochemical detection of bisphenol S based on 1T&2H-MoS_2_/CNTs-NH_2_ nanocomposites. New J. Chem..

[35] Tan C., Zhang H. (2015). Wet-chemical synthesis and applications of non-layer structured two-dimensional nanomaterials. Nat. Commun..

[36] Joshi R.K., Schneider J.J. (2012). Assembly of one dimensional inorganic nanostructures into functional 2D and 3D architectures. Synthesis, arrangement and functionality. Chem. Soc. Rev..

[37] Wang J., Ma C., Su L., Gong L., Dong D., Wu Z. (2020). Self-Assembly/Sacrificial Synthesis of Highly Capacitive Hierarchical Porous Carbon from Longan Pulp Biomass. ChemElectroChem.

[38] Xu J., Ma Y., Xuan C., Ma C., Wang J. (2022). Three-Dimensional Electrodes for Oxygen Electrocatalysis. ChemElectroChem.

[39] Jiang J., Li Y., Liu J., Huang X., Yuan C., Lou X.W. (2012). Recent advances in metal oxide-based electrode architecture design for electrochemical energy storage. Adv. Mater..

[40] Liu M., Dong H., Zhang S., Chen X., Sun Y., Gao S., Xu J., Wu X., Yuan A., Lu W. (2019). Three-Dimensional Porous TiNb_2_O_7_/CNT-KB Composite Microspheres as Lithium-Ion Battery Anode Material. ChemElectroChem.

[41] Yu M., Qiu W., Wang F., Zhai T., Fang P., Lu X., Tong Y. (2015). Three dimensional architectures: Design, assembly and application in electrochemical capacitors. J. Mater. Chem. A.

[42] Zhang H., Ning H., Busbee J., Shen Z., Kiggins C., Hua Y., Eaves J., Davis J., Shi T., Shao Y.-T. (2017). Electroplating lithium transition metal oxides. Sci. Adv..

[43] Pu J., Shen Z., Zhong C., Zhou Q., Liu J., Zhu J., Zhang H. (2020). Electrodeposition Technologies for Li-Based Batteries: New Frontiers of Energy Storage. Adv. Mater..

[44] Beidaghi M., Gogotsi Y. (2014). Capacitive energy storage in micro-scale devices: Recent advances in design and fabrication of micro-supercapacitors. Energy Environ. Sci..

[45] Singh M., Ghosh R., Chen Y.-S., Yen Z.-L., Hofmann M., Chen Y.-F., Hsieh Y.-P. (2022). Chemical vapor deposition merges MoS2 grains into high-quality and centimeter-scale films on Si/SiO_2_. RSC Adv..

[46] Gerard O., Numan A., Krishnan S., Khalid M., Subramaniam R., Kasi R. (2022). A review on the recent advances in binder-free electrodes for electrochemical energy storage application. J. Energy Storage.

[47] Shao Z., Chang Y., Venton B.J. (2022). Carbon microelectrodes with customized shapes for neurotransmitter detection: A review. Anal. Chim. Acta.

[48] Banciu C.A., Nastase F., Istrate A.-I., Veca L.M. (2022). 3D Graphene Foam by Chemical Vapor Deposition: Synthesis, Properties, and Energy-Related Applications. Molecules.

[49] Chen K., Chai Z., Li C., Shi L., Liu M., Xie Q., Zhang Y., Xu D., Manivannan A., Liu Z. (2016). Catalyst-Free Growth of Three-Dimensional Graphene Flakes and Graphene/g-C_3_N_4_ Composite for Hydrocarbon Oxidation. ACS Nano.

[50] Jayasinghe C., Chakrabarti S., Schulz M.J., Shanov V. (2011). Spinning yarn from long carbon nanotube arrays. J. Mater. Res..

[51] Kumar A., Nanda D., Samal S.K., Mohanty S., Nayak S.K. (2019). Chapter 3—Methods and fabrication techniques of superhydrophobic surfaces. Superhydrophobic Polymer Coatings.

[52] Hamedani Y., Macha P., Bunning T.J., Naik R.R., Vasudev M.C., Sudheer N. (2016). Plasma-Enhanced Chemical Vapor Deposition: Where we are and the Outlook for the Future. Chemical Vapor Deposition.

[53] Vernardou D. (2021). Progress and Challenges in Industrially Promising Chemical Vapour Deposition Processes for the Synthesis of Large-Area Metal Oxide Electrode Materials Designed for Aqueous Battery Systems. Materials.

[54] Yu L., Wu H.B., Lou X.W. (2013). Mesoporous Li_4_Ti_5_O_12_ Hollow Spheres with Enhanced Lithium Storage Capability. Adv. Mater..

[55] Khalily M.A., Eren H., Akbayrak S., Susapto H.H., Biyikli N., Özkar S., Guler M.O. (2016). Facile Synthesis of Three-Dimensional Pt-TiO_2_ Nano-networks: A Highly Active Catalyst for the Hydrolytic Dehydrogenation of Ammonia–Borane. Angew. Chem. Int. Ed..

[56] Wu H.B., Pan A., Hng H.H., Lou X.W. (2013). Template-Assisted Formation of Rattle-type V_2_O_5_ Hollow Microspheres with Enhanced Lithium Storage Properties. Adv. Funct. Mater..

[57] Miao J., Lang Z., Xue T., Li Y., Li Y., Cheng J., Zhang H., Tang Z. (2020). Revival of Zeolite-Templated Nanocarbon Materials: Recent Advances in Energy Storage and Conversion. Adv. Sci..

[58] Sivakumar M., Muthukutty B., Chen T.W., Chen S.M., Vivekanandan A.K., Chen S.H., Hatshan M.R., Ali M.A., Kumar M. (2022). Electrocatalytic detection of noxious antioxidant diphenylamine in fruit samples with support of Cu@nanoporous carbon modified sensor. Chemosphere.

[59] Sohrabi H., Salahshour Sani P., Orooji Y., Majidi M.R., Yoon Y., Khataee A. (2022). MOF-based sensor platforms for rapid detection of pesticides to maintain food quality and safety. Food Chem. Toxicol..

[60] Chao D., Xia X., Liu J., Fan Z., Ng C.F., Lin J., Zhang H., Shen Z.X., Fan H.J. (2014). A V_2_O_5_/Conductive-Polymer Core/Shell Nanobelt Array on Three-Dimensional Graphite Foam: A High-Rate, Ultrastable, and Freestanding Cathode for Lithium-Ion Batteries. Adv. Mater..

[61] Liu Y., Goebl J., Yin Y. (2013). Templated synthesis of nanostructured materials. Chem. Soc. Rev..

[62] Wu B., Hou S., Miao Z., Zhang C., Ji Y. (2015). Layer-by-Layer Self-Assembling Gold Nanorods and Glucose Oxidase onto Carbon Nanotubes Functionalized Sol-Gel Matrix for an Amperometric Glucose Biosensor. Nanomaterials.

[63] Kravanja K.A., Finšgar M. (2022). A review of techniques for the application of bioactive coatings on metal-based implants to achieve controlled release of active ingredients. Mater. Des..

[64] He W., Liu P.-J., He G.-Q., Gozin M., Yan Q.-L. (2018). Highly Reactive Metastable Intermixed Composites (MICs): Preparation and Characterization. Adv. Mater..

[65] Parra-Alfambra A.M., Casero E., Petit-Domínguez M.D., Barbadillo M., Pariente F., Vázquez L., Lorenzo E. (2011). New nanostructured electrochemical biosensors based on three-dimensional (3-mercaptopropyl)-trimethoxysilane network. Analyst.

[66] Yan D., Meng Z.-H., Qiu L.-L., Xue M. (2021). Recent Advances in Preparation and Applications of 3D Transition Metal Oxides Semiconductor Photonic Crystal. Adv. Photonics Res..

[67] Vinoth Kumar J., Karthik R., Chen S.-M., Chen K.-H., Sakthinathan S., Muthuraj V., Chiu T.-W. (2018). Design of novel 3D flower-like neodymium molybdate: An efficient and challenging catalyst for sensing and destroying pulmonary toxicity antibiotic drug nitrofurantoin. Chem. Eng. J..

[68] Hou S., Ou Z., Chen Q., Wu B. (2012). Amperometric acetylcholine biosensor based on self-assembly of gold nanoparticles and acetylcholinesterase on the sol–gel/multi-walled carbon nanotubes/choline oxidase composite-modified platinum electrode. Biosens. Bioelectron..

[69] He J., Wang S., Jiang L., Li X., Hong Q., Zhu W., Sun J., Zhang X., Xu Z. (2022). Femtosecond Laser One-Step Direct Writing Electrodes with Ag NPs-Graphite Carbon Composites for Electrochemical Sensing. Adv. Mater. Technol..

[70] Ansari A.A., Solanki P.R., Malhotra B.D. (2008). Sol-gel derived nanostructured cerium oxide film for glucose sensor. Appl. Phys. Lett..

[71] Zhao D.-D., Bao S.-J., Zhou W.-J., Li H.-L. (2007). Preparation of hexagonal nanoporous nickel hydroxide film and its application for electrochemical capacitor. Electrochem. Commun..

[72] Wang G., Zhang L., Zhang J. (2012). A review of electrode materials for electrochemical supercapacitors. Chem. Soc. Rev..

[73] Solanki P.R., Kaushik A., Agrawal V.V., Malhotra B.D. (2011). Nanostructured metal oxide-based biosensors. NPG Asia Mater..

[74] Wang C., Du L., Xing X., Feng D., Tian Y., Li Z., Yang D. (2022). Flexible carbon cloth in-situ assembling WO_3_ microsheets bunches with Ni dopants for non-enzymatic glucose sensing. Appl. Surf. Sci..

[75] Vinoth S., Wang S.-F. (2023). Construction of functionalized carbon nanotube@metal oxide nanocomposite for high-performance electrochemical measurement of antipyretic drug in water samples. Environ. Sci. Pollut. Res..

[76] Nguyen N.S., Das G., Yoon H.H. (2016). Nickel/cobalt oxide-decorated 3D graphene nanocomposite electrode for enhanced electrochemical detection of urea. Biosens. Bioelectron..

[77] Zhang K., Zeng H., Feng J., Liu Z., Chu Z., Jin W. (2022). Screen-printing of core-shell Mn3O4@C nanocubes based sensing microchip performing ultrasensitive recognition of allura red. Food Chem. Toxicol..

[78] Marimuthu M., Arumugam S.S., Jiao T., Sabarinathan D., Li H., Chen Q. (2022). Metal organic framework based sensors for the detection of food contaminants. TrAC Trends Anal. Chem..

[79] Wang S., McGuirk C.M., d’Aquino A., Mason J.A., Mirkin C.A. (2018). Metal-Organic Framework Nanoparticles. Adv. Mater..

[80] Férey G. (2008). Hybrid porous solids: Past, present, future. Chem. Soc. Rev..

[81] Iftikhar T., Aziz A., Ashraf G., Xu Y., Li G., Zhang T., Asif M., Xiao F., Liu H. (2022). Engineering MOFs derived metal oxide nanohybrids: Towards electrochemical sensing of catechol in tea samples. Food Chem..

[82] Chen X., Li J., Li J., Zhang L., Zhao P., Wang C., Fei J., Xie Y. (2022). Determination of luteolin in Chrysanthemum tea with a ultra-sensitive electrochemical sensor based on MoO_3_/poly(3,4-ethylene dioxythiophene)/gama-cyclodextrin metal-organic framework composites. Food Chem..

[83] Hitabatuma A., Wang P., Su X., Ma M. (2022). Metal-Organic Frameworks-Based Sensors for Food Safety. Foods.

[84] Sindoro M., Yanai N., Jee A.-Y., Granick S. (2014). Colloidal-Sized Metal–Organic Frameworks: Synthesis and Applications. Acc. Chem. Res..

[85] Tanabe K.K., Cohen S.M. (2011). Postsynthetic modification of metal–organic frameworks—A progress report. Chem. Soc. Rev..

[86] Sakata Y., Furukawa S., Kondo M., Hirai K., Horike N., Takashima Y., Uehara H., Louvain N., Meilikhov M., Tsuruoka T. (2013). Shape-Memory Nanopores Induced in Coordination Frameworks by Crystal Downsizing. Science.

[87] LaMer V.K., Dinegar R.H. (1950). Theory, Production and Mechanism of Formation of Monodispersed Hydrosols. J. Am. Chem. Soc..

[88] Haque E., Khan N.A., Park J.H., Jhung S.H. (2010). Synthesis of a Metal–Organic Framework Material, Iron Terephthalate, by Ultrasound, Microwave, and Conventional Electric Heating: A Kinetic Study. Chem. Eur. J..

[89] Hermes S., Witte T., Hikov T., Zacher D., Bahnmüller S., Langstein G., Huber K., Fischer R.A. (2007). Trapping Metal-Organic Framework Nanocrystals:  An in-Situ Time-Resolved Light Scattering Study on the Crystal Growth of MOF-5 in Solution. J. Am. Chem. Soc..

[90] Avci C., Ariñez-Soriano J., Carné-Sánchez A., Guillerm V., Carbonell C., Imaz I., Maspoch D. (2015). Post-Synthetic Anisotropic Wet-Chemical Etching of Colloidal Sodalite ZIF Crystals. Angew. Chem. Int. Ed..

[91] Lu X., He B., Liang Y., Wang J., Jiao Q., Liu Y., Guo R., Wei M., Jin H., Ren W. (2022). An electrochemical aptasensor based on dual-enzymes-driven target recycling strategy for patulin detection in apple juice. Food Control.

[92] Yeung H.H.M., Sapnik A.F., Massingberd-Mundy F., Gaultois M.W., Wu Y., Fraser D.A.X., Henke S., Pallach R., Heidenreich N., Magdysyuk O.V. (2019). Control of Metal–Organic Framework Crystallization by Metastable Intermediate Pre-equilibrium Species. Angew. Chem. Int. Ed..

[93] Zavyalova A.G., Kladko D.V., Chernyshov I.Y., Vinogradov V.V. (2021). Large MOFs: Synthesis strategies and applications where size matters. J. Mater. Chem. A.

[94] Chen Y., Yang Z., Hu H., Zhou X., You F., Yao C., Liu F.J., Yu P., Wu D., Yao J. (2022). Advanced Metal-Organic Frameworks-Based Catalysts in Electrochemical Sensors. Front. Chem..

[95] Wang Y., Wang L., Huang W., Zhang T., Hu X., Perman J.A., Ma S. (2017). A metal–organic framework and conducting polymer based electrochemical sensor for high performance cadmium ion detection. J. Mater. Chem. A.

[96] Lv L., Zhang B., Tian P., Xie L., Wei W., He J., Lin M., Zhu H., Chen H., He B. (2022). A “signal off” aptasensor based on AuNPs/Ni-MOF substrate-free catalyzed for detection Enrofloxacin. J. Electroanal. Chem..

[97] Xu Z., Li P., Chen H., Zhu X., Zhang Y., Liu M., Yao S. (2022). Picomolar glutathione detection based on the dual-signal self-calibration electrochemical sensor of ferrocene-functionalized copper metal-organic framework via solid-state electrochemistry of cuprous chloride. J. Colloid Interface Sci..

[98] Khataee A., Sohrabi H., Ehsani M., Agaei M., Sisi A.J., Abdi J., Yoon Y. (2022). State-of-the-art progress of metal-organic framework-based electrochemical and optical sensing platforms for determination of bisphenol A as an endocrine disruptor. Environ. Res..

[99] Peng C., Miao L., Qiu D., Chen S. (2022). Co_3_O_4_-chitosan/biomass-derived porous carbon molecularly imprinted polymer integrated electrode for selective detection of glucose. Ceram. Int..

[100] Moradi O. (2022). Electrochemical sensors based on carbon nanostructures for the analysis of bisphenol A—A review. Food Chem. Toxicol..

[101] Wu Z.Y., Liang H.W., Chen L.F., Hu B.C., Yu S.H. (2016). Bacterial Cellulose: A Robust Platform for Design of Three Dimensional Carbon-Based Functional Nanomaterials. Acc. Chem. Res..

[102] Li C., Shi G. (2012). Three-dimensional graphene architectures. Nanoscale.

[103] Dong X.-C., Xu H., Wang X.-W., Huang Y.-X., Chan-Park M.B., Zhang H., Wang L.-H., Huang W., Chen P. (2012). 3D Graphene–Cobalt Oxide Electrode for High-Performance Supercapacitor and Enzymeless Glucose Detection. ACS Nano.

[104] Deline A.R., Frank B.P., Smith C.L., Sigmon L.R., Wallace A.N., Gallagher M.J., Goodwin D.G., Durkin D.P., Fairbrother D.H. (2020). Influence of Oxygen-Containing Functional Groups on the Environmental Properties, Transformations, and Toxicity of Carbon Nanotubes. Chem. Rev..

[105] Si X., Deng L., Wang Y., Han M., Ding Y. (2022). An electrochemical sensor for the determination of Luteolin using an alizarin red/carboxylic acid group functionalized carbon nanotube. Microchem. J..

[106] Xuan X., Kim J.Y., Hui X., Das P.S., Yoon H.S., Park J.-Y. (2018). A highly stretchable and conductive 3D porous graphene metal nanocomposite based electrochemical-physiological hybrid biosensor. Biosens. Bioelectron..

[107] Chen Y., Zhao F., Zeng B. (2022). Fabrication of surface molecularly imprinted electrochemical sensor for the sensitive quantification of chlortetracycline with ionic liquid and MWCNT improving performance. Talanta.

[108] Liu R., Chang Y., Li F., Dubovyk V., Li D., Ran Q., Zhao H. (2022). Highly sensitive detection of carbendazim in juices based on mung bean-derived porous carbon@chitosan composite modified electrochemical sensor. Food Chem..

[109] Liu W., Yang X., Li M., Gui Q.-W., Jiang H., Li Y., Shen Q., Xia J., Liu X. (2022). Sensitive detection of luteolin in peanut shell based on titanium carbide/carbon nanotube composite modified screen-printed electrode. Microchem. J..

[110] Liu R., Li B., Li F., Dubovyk V., Chang Y., Li D., Ding K., Ran Q., Wang G., Zhao H. (2022). A novel electrochemical sensor based on beta-cyclodextrin functionalized carbon nanosheets@carbon nanotubes for sensitive detection of bactericide carbendazim in apple juice. Food Chem..

[111] Chen Y., Tang Y., Liu Y., Zhao F., Zeng B. (2022). Kill two birds with one stone: Selective and fast removal and sensitive determination of oxytetracycline using surface molecularly imprinted polymer based on ionic liquid and ATRP polymerization. J. Hazard. Mater..

[112] Zhang K., Wang Y., Wang H., Li F., Zhang Y., Zhang N. (2022). Three-dimensional porous reduced graphene oxide modified electrode for highly sensitive detection of trace rifampicin in milk. Anal. Methods.

[113] Karuppiah C., Babulal S.M., Chen T.-W., Chen S.-M., Hsu L.-F., Al Farraj D.A., Ramaraj S.K., Elshikh M.S., Yang C.-C. (2022). A novel ammonium zinc molybdate layered double hydroxide nanoflakes/vapor grown carbon fibers nanomaterials based electrocatalyst for the monitoring of dimetridazole drug in real samples. J. Environ. Chem. Eng..

[114] Priscillal I.J.D., Wang S.F. (2022). Synchronously activated strontium aluminate nanoflakes anchored functionalized carbon nanofiber nanocomposite for sensitive amperometric detection of food additive: Propyl gallate. Food Chem..

[115] Lu L., Kang J. (2018). Amperometric nonenzymatic sensing of glucose at very low working potential by using a nanoporous PdAuNi ternary alloy. Microchim. Acta.

[116] Lu L. (2019). Nanoporous noble metal-based alloys: A review on synthesis and applications to electrocatalysis and electrochemical sensing. Mikrochim. Acta.

[117] Weremfo A., Fong S.T.C., Khan A., Hibbert D.B., Zhao C. (2017). Electrochemically roughened nanoporous platinum electrodes for non-enzymatic glucose sensors. Electrochim. Acta.

[118] Xu Y., Zhang B. (2014). Recent advances in porous Pt-based nanostructures: Synthesis and electrochemical applications. Chem. Soc. Rev..

[119] Li C., Iqbal M., Lin J., Luo X., Jiang B., Malgras V., Wu K.C.W., Kim J., Yamauchi Y. (2018). Electrochemical Deposition: An Advanced Approach for Templated Synthesis of Nanoporous Metal Architectures. Acc. Chem. Res..

[120] Li C., Eid K., Wang H., Deng Y., Lu S., Li X., Wang L., Gu H. (2018). One-pot synthesis of bimetallic PdCu nanoframes as an efficient catalyst for the methanol oxidation reaction. New J. Chem..

[121] Thanh T.D., Balamurugan J., Lee S.H., Kim N.H., Lee J.H. (2016). Novel porous gold-palladium nanoalloy network-supported graphene as an advanced catalyst for non-enzymatic hydrogen peroxide sensing. Biosens. Bioelectron..

[122] Zhao D., Xu C. (2015). A nanoporous palladium-nickel alloy with high sensing performance towards hydrogen peroxide and glucose. J. Colloid Interface Sci..

[123] Zhao D., Yu G., Tian K., Xu C. (2016). A highly sensitive and stable electrochemical sensor for simultaneous detection towards ascorbic acid, dopamine, and uric acid based on the hierarchical nanoporous PtTi alloy. Biosens. Bioelectron..

[124] Celik Kazici H., Caglar A., Aydogmus T., Aktas N., Kivrak H. (2018). Microstructured prealloyed Titanium-Nickel powder as a novel nonenzymatic hydrogen peroxide sensor. J. Colloid Interface Sci..

[125] Kang Y., Jiang B., Alothman Z.A., Badjah A.Y., Naushad M., Habila M., Wabaidur S., Henzie J., Li H., Yamauchi Y. (2019). Mesoporous PtCu Alloy Nanoparticles with Tunable Compositions and Particles Sizes Using Diblock Copolymer Micelle Templates. Chem. Eur. J..

[126] Sivakumar M., Pandi K., Chen S.-M., Cheng Y.-H., Sakthivel M. (2017). Facile synthesis of perovskite-type NdNiO_3_ nanoparticles for an effective electrochemical non-enzymatic glucose biosensor. New J. Chem..

[127] Boubezari I., Zazoua A., Errachid A., Jaffrezic-Renault N. (2021). Sensitive Electrochemical Detection of Bioactive Molecules (Hydrogen Peroxide, Glucose, Dopamine) with Perovskites-Based Sensors. Chemosensors.

[128] Govindasamy M., Wang S.-F., Huang C.-H., Alshgari R.A., Ouladsmane M. (2022). Colloidal synthesis of perovskite-type lanthanum aluminate incorporated graphene oxide composites: Electrochemical detection of nitrite in meat extract and drinking water. Microchim. Acta.

[129] Wang Y.-Z., Zhong H., Li X.-M., Jia F.-F., Shi Y.-X., Zhang W.-G., Cheng Z.-P., Zhang L.-L., Wang J.-K. (2013). Perovskite LaTiO_3_–Ag0.2 nanomaterials for nonenzymatic glucose sensor with high performance. Biosens. Bioelectron..

[130] Ali S.M., Al-Otaibi H.M. (2019). The distinctive sensing performance of cobalt ion in LaBO_3_ perovskite (B = Fe, Mn, Ni, or Cr) for hydrazine electrooxidation. J. Electroanal. Chem..

[131] Tiliakos A., Ceaus C., Iordache S.M., Vasile E., Stamatin I. (2016). Morphic transitions of nanocarbons via laser pyrolysis of polyimide films. J. Anal. Appl. Pyrolysis.

[132] Wang H., Zhu W., Xu T., Zhang Y., Tian Y., Liu X., Wang J., Ma M. (2022). An integrated nanoflower-like MoS_2_@CuCo_2_O_4_ heterostructure for boosting electrochemical glucose sensing in beverage. Food Chem..

[133] Yan L., Chu D., Chu X.-Q., Ge D., Chen X. (2022). Co/CoO nanoparticles armored by N-doped nanoporous carbon polyhedrons towards glucose oxidation in high-performance non-enzymatic sensors. New J. Chem..

[134] Li M., Fang L., Zhou H., Wu F., Lu Y., Luo H., Zhang Y., Hu B. (2019). Three-dimensional porous MXene/NiCo-LDH composite for high performance non-enzymatic glucose sensor. Appl. Surf. Sci..

[135] Thenrajan T., Selvasundarasekar S.S., Kundu S., Wilson J. (2022). Novel Electrochemical Sensing of Catechins in Raw Green Tea Extract via a Trimetallic Zeolitic Imidazolate Fibrous Framework. ACS Omega.

[136] Tang J., Hu T., Li N., Zhu Y., Li J., Zheng S., Guo J. (2022). Ag doped Co/Ni bimetallic organic framework for determination of luteolin. Microchem. J..

[137] Balram D., Lian K.Y., Sebastian N., Al-Mubaddel F.S., Noman M.T. (2022). Ultrasensitive detection of food colorant sunset yellow using nickel nanoparticles promoted lettuce-like spinel Co_3_O_4_ anchored GO nanosheets. Food Chem. Toxicol..

[138] Garkani Nejad F., Asadi M.H., Sheikhshoaie I., Dourandish Z., Zaimbashi R., Beitollahi H. (2022). Construction of modified screen-printed graphite electrode for the application in electrochemical detection of sunset yellow in food samples. Food Chem. Toxicol..

[139] Chen Y., Waterhouse G.I.N., Sun H., Qiao X., Sun Y., Xu Z. (2022). Novel ratiometric electrochemical sensing platform with dual-functional poly-dopamine and NiS@HCS signal amplification for sunset yellow detection in foods. Food Chem..

[140] Joseph X.B., Sherlin V.A., Wang S.F., George M. (2022). Integration of iron-manganese layered double hydroxide/tungsten carbide composite: An electrochemical tool for diphenylamine H(*+) analysis in environmental samples. Environ. Res..

[141] Ghalkhani M., Gharagozlou M., Sohouli E., Marzi Khosrowshahi E. (2022). Preparation of an electrochemical sensor based on a HKUST-1/CoFe_2_O_4_/SiO_2_-modified carbon paste electrode for determination of azaperone. Microchem. J..

[142] Xia Y., Hu X., Liu Y., Zhao F., Zeng B. (2022). Molecularly imprinted ratiometric electrochemical sensor based on carbon nanotubes/cuprous oxide nanoparticles/titanium carbide MXene composite for diethylstilbestrol detection. Mikrochim. Acta.

[143] Keerthi M., Kumar Panda A., Wang Y.H., Liu X., He J.H., Chung R.J. (2022). Titanium nanoparticle anchored functionalized MWCNTs for electrochemical detection of ractopamine in porcine samples with ultrahigh sensitivity. Food Chem..

[144] Lei Y., Zhang Y., Yuan L., Li J. (2022). Biochar-supported Cu nanocluster as an electrochemical ultrasensitive interface for ractopamine sensing. Food Chem. X.

[145] Ma F., Li X., Li Y., Feng Y., Ye B.C. (2022). High current flux electrochemical sensor based on nickel-iron bimetal pyrolytic carbon material of paper waste pulp for clenbuterol detection. Talanta.

[146] Radhakrishnan S., Mathiyarasu J., Kim B.-S. (2022). Environmental-assisted shape-controlled synthesis and electrocatalytic performance of CuS nanostructures for vanillin detection in commercial food products. Appl. Mater. Today.

[147] Kogularasu S., Sriram B., Wang S.-F., Sheu J.-K. (2022). Sea-Urchin-Like Bi2S3 Microstructures Decorated with Graphitic Carbon Nitride Nanosheets for Use in Food Preservation. ACS Appl. Nano Mater..

[148] Cheng Z., Song H., Zhang X., Cheng X., Xu Y., Zhao H., Gao S., Huo L. (2022). Non-enzymatic nitrite amperometric sensor fabricated with near-spherical ZnO nanomaterial. Colloids Surf. B Biointerfaces.

[149] Somnet K., Soravech P., Karuwan C., Tuantranont A., Amatatongchai M. (2022). A compact N-nitrosodiphenylamine imprinted sensor based on a Pd nanoparticles-MIP microsphere modified screen-printed graphene electrode. J. Electroanal. Chem..

[150] Lu H., Wang H., Yang L., Zhou Y., Xu L., Hui N., Wang D. (2021). A sensitive electrochemical sensor based on metal cobalt wrapped conducting polymer polypyrrole nanocone arrays for the assay of nitrite. Mikrochim. Acta.

[151] Han S., Ding Y., Teng F., Yao A., Leng Q. (2022). Molecularly imprinted electrochemical sensor based on 3D-flower-like MoS_2_ decorated with silver nanoparticles for highly selective detection of butylated hydroxyanisole. Food Chem..

[152] Tang J., Li J., Liu T., Tang W., Li N., Zheng S., Guo J., Song C. (2022). N-Doped TiO_2_–Carbon Composites Derived from NH_2_-MIL-_125_(Ti) for Electrochemical Determination of tert-Butylhydroquinone. Food Anal. Methods.

[153] Luo Y., Yang Y., Wang L., Wang L., Chen S. (2022). An ultrafine ZnO/ZnNi_2_O_4_@porous carbon@covalent-organic framework for electrochemical detection of paracetamol and tert-butyl hydroquinone. J. Alloys Compd..

[154] Balram D., Lian K.-Y., Sebastian N., Al-Mubaddel F.S., Noman M.T. (2022). A sensitive and economical electrochemical platform for detection of food additive tert-butylhydroquinone based on porous Co_3_O_4_ nanorods embellished chemically oxidized carbon black. Food Control.

[155] Ru J., Wang X., Zhou Z., Zhao J., Yang J., Du X., Lu X. (2022). Fabrication of octahedral GO/UiO-67@PtNPs nanocomposites as an electrochemical sensor for ultrasensitive recognition of arsenic (III) in Chinese Herbal Medicine. Anal Chim Acta.

[156] Liu T., Lin B., Yuan X., Chu Z., Jin W. (2022). In situ fabrication of urchin-like Cu@carbon nanoneedles based aptasensor for ultrasensitive recognition of trace mercury ion. Biosens. Bioelectron..

[157] Ganesan M., Keerthika Devi R., Liao A.H., Lee K.Y., Gopalakrishnan G., Chuang H.C. (2022). 3D-flower-like porous neodymium molybdate nanostructure for trace level detection of organophosphorus pesticide in food samples. Food Chem..

[158] Su X., Chen Z., Wang H., Yuan L., Zheng K., Zhang W., Zou X. (2022). Ratiometric immunosensor with DNA tetrahedron nanostructure as high-performance carrier of reference signal and its applications in selective phoxim determination for vegetables. Food Chem..

[159] Mahmoudi-Moghaddam H., Akbari Javar H., Garkani-Nejad Z. (2022). Fabrication of platinum-doped NiCo_2_O_4_ nanograss modified electrode for determination of carbendazim. Food Chem..

[160] Tamilalagan E., Akilarasan M., Chen S.-M., Maheshwaran S., Huang Y.-F. (2022). Rationally designed urchin-like structured cobalt diselenide (o-CoSe_2_) for the sensitive voltammetric detection of carbendazim fungicide in vegetables and water samples. Colloids Surf. A Physicochem. Eng. Asp..

[161] Akilarasan M., Maheshwaran S., Chen S.-M., Tamilalagan E., Albaqami M.D., Alotabi R.G., Arumugam R. (2022). In-situ synthesis of bimetallic chalcogenide SrS/Bi_2_S_3_ nanocomposites as an efficient electrocatalyst for the selective voltammetric sensing of maleic hydrazide herbicide. Process Saf. Environ. Prot..

[162] Yamuna A., Karikalan N., Lee T.Y. (2022). Effect of the Ni3TeO6 phase in a Ni2Te3O8/expanded graphite composite on the electrochemical monitoring of metribuzin residue in soil and water samples. J. Hazard. Mater..

[163] Manjula N., Pulikkutty S., Chen S.-M. (2022). Hexagonal plate-like NiO/ZnO for highly selective detection of antibiotic drugs in food and biological samples. FlatChem.

[164] Li M., Zhe T., Li F., Li R., Bai F., Jia P., Bu T., Xu Z., Wang L. (2022). Hybrid structures of cobalt-molybdenum bimetallic oxide embedded in flower-like molybdenum disulfide for sensitive detection of the antibiotic drug nitrofurantoin. J. Hazard. Mater..

[165] Zou F., Fu K., Jin C., Li M., Zhang G., Zhang R., Bai H. (2022). Microwave-prepared surface imprinted magnetic nanoparticles based electrochemical sensor for adsorption and determination of ketamine in sewage. Anal. Chim. Acta.

[166] Roushani M., Farokhi S., Rahmati Z. (2022). Development of a dual-recognition strategy for the aflatoxin B1 detection based on a hybrid of aptamer-MIP using a Cu_2_O NCs/GCE. Microchem. J..

[167] Feng K., Li T., Ye C., Gao X., Yue X., Ding S., Dong Q., Yang M., Huang G., Zhang J. (2022). A novel electrochemical immunosensor based on Fe_3_O_4_@graphene nanocomposite modified glassy carbon electrode for rapid detection of Salmonella in milk. J. Dairy Sci..

[168] Fatema K.N., Areerob Y., Meng Z.-D., Zhu L., Oh W.-C. (2022). Cu- and Sn-Codoped Mesoporous BaTiO_3_-G-SiO_2_ Nanocomposite for Bioreceptor-Free, Sensitive, and Quick Electrochemical Sensing of Rhizopus stolonifer Fungus. ACS Appl. Electron. Mater..

[169] Lai Q., Niu Q., Chen W., Zhang Y., Long M., Liang B., Wang F., Liu Z. (2022). An ultrasensitive bacteria biosensor using “multilayer cake” silver microelectrode based on local high electric field effect. Appl. Phys. Lett..

[170] Zheng R., He B., Xie L., Yan H., Jiang L., Ren W., Suo Z., Xu Y., Wei M., Jin H. (2022). Molecular Recognition-Triggered Aptazyme Sensor Using a Co-MOF@MCA Hybrid Nanostructure as Signal Labels for Adenosine Triphosphate Detection in Food Samples. Anal. Chem..

